# Exosomal miR-3682-3p Suppresses Angiogenesis by Targeting ANGPT1 via the RAS-MEK1/2-ERK1/2 Pathway in Hepatocellular Carcinoma

**DOI:** 10.3389/fcell.2021.633358

**Published:** 2021-03-29

**Authors:** Shuang-Shuang Dong, Dan-Dan Dong, Zhang-Fu Yang, Gui-Qi Zhu, Dong-Mei Gao, Jie Chen, Yan Zhao, Bin-Bin Liu

**Affiliations:** ^1^Liver Cancer Institute, Zhongshan Hospital, Fudan University and Key Laboratory of Carcinogenesis and Cancer Invasion, Ministry of Education, Shanghai, China; ^2^Department of Biochemistry and Molecular Biology, School of Basic Medical Sciences, Fudan University, Shanghai, China

**Keywords:** HCC, exosomes, angiogenesis, miR-3682-3p, ANGPT1

## Abstract

**Background:**

Angiogenesis is a crucial process in tumorigenesis and development. The role of exosomes derived from hepatocellular carcinoma (HCC) cells in angiogenesis has not been clearly elucidated.

**Methods and Results:**

Exosomes were isolated from HCC cell lines (HCCLM3, MHCC97L, and PLC/RFP/5) by ultracentrifugation and identified by nano transmission electron microscopy (TEM), NanoSight analysis and western blotting, respectively. *In vitro* and *in vivo* analyses showed that exosomes isolated from highly metastatic HCC cells enhanced the migration, invasion and tube formation of human umbilical vein endothelial cells (HUVECs) compared to exosomes derived from poorly metastatic HCC cells. In addition, microarray analysis of HCC-Exos was conducted to identify potential functional molecules, and miR-3682-3p expression was found to be significantly downregulated in exosomes isolated from highly metastatic HCC cells. By *in vitro* gain-of-function experiments, we found that HCC cells secreted exosomal miR-3682-3p, which negatively regulates angiopoietin-1 (ANGPT1), and this led to inhibition of RAS-MEK1/2-ERK1/2 signaling in endothelial cells and eventually impaired angiogenesis.

**Conclusion:**

Our study elucidates that exosomal miR-3682-3p attenuates angiogenesis by targeting ANGPT1 through RAS-MEK1/2-ERK1/2 signaling and provides novel potential targets for liver cancer therapy.

## Introduction

Liver cancer is one of the most common malignancies, ranking sixth globally in incidence and fourth in mortality (Bray et al., 2018). Hepatocellular carcinoma (HCC) is the most common type of primary liver cancer, accounting for approximately 750,000 deaths worldwide each year, and more than 50% of the cases occur in China (Siegel et al., 2018). Despite progress in treatment, such as surgical resection, radiofrequency thermal ablation, liver transplantation, and adjuvant therapy, the prognosis of HCC patients remains poor due to high recurrence and metastasis rates (Zhu et al., 2019). Therefore, identification of novel therapeutic targets and further exploration of the molecular mechanisms underlying HCC are urgently needed.

Exosomes (Exos) are lipid bilayer-enclosed vesicles with a diameter of 30–200 nm (Pegtel and Gould, 2019), and they are secreted by almost all cells. Specific proteins are highly expressed in Exos, such as Alix, CD9, CD81, and TSG101, which are usually used as markers for the identification of exosomes (Pfeffer, 2010). Increasing evidence has demonstrated that exosomes play important roles in the regulation of the tumor microenvironment via the promotion of angiogenesis, signaling pathway activation, tumorigenesis, and metastasis (Taylor and Gercel-Taylor, 2011). Exosomes contain many functional molecules, such as proteins, DNA, RNA, and lipids, and play important roles in intercellular material and information transmission.

Angiogenesis, the generation of new blood vessels, is one of the hallmarks of cancer because it plays essential roles in tumor cell growth, invasion, recurrence, and metastasis by supplying oxygen and nutrients as well as transporting carbon dioxide and metabolic wastes (Hanahan and Weinberg, 2011). It has been widely shown that active angiogenesis is responsible for the rapid growth of tumor cells and poor prognosis of cancer patients (Fang et al., 2011). Although the mechanism of tumor angiogenesis has been studied for decades, the detailed mechanism and crosstalk between effector and recipient cells have not been well elucidated. The most well-studied molecule implicated in tumor angiogenesis is vascular endothelial growth factor (VEGF) (Goel and Mercurio, 2013); however, other involved molecules still require further study. Angiopoietin-1 (ANGPT1) is a member of the human ANGPT-TIE protein family, which includes three ligands (ANGPT1, ANGPT2, and ANGPT4) and two receptors (TIE1 and TIE2). ANGPT1 is considered to be the most important mediator of the VEGF-independent proangiogenic signaling pathway. Extensive investigations into its roles in the process of tumor angiogenesis are still ongoing.

MicroRNAs (miRNAs) are small, endogenous, non-coding RNAs of 18-24 nucleotides that are implicated in the posttranscriptional regulation of gene expression (Ediriweera and Cho, 2019). They bind to complementary sites within the 3′ untranslated region (UTR) or open reading frame to target messenger RNAs (mRNAs), leading to downregulation of protein expression or degradation of the target mRNA (Iwakawa and Tomari, 2013). Emerging evidence indicates that miRNAs can be selectively packed into extracellular vesicles, particularly exosomes, secreted into the extracellular fluid, and delivered into recipient cells to affect a variety of biological processes, such as differentiation, proliferation, and apoptosis (De Bem et al., 2017). However, the relation between angiogenesis and HCC cell-derived exosomal miRNAs has not been clearly elucidated.

In our study, we found that HCC-derived exosomes promoted angiogenesis *in vitro* and *in vivo*. Furthermore, we examined the exosomal miRNA expression profiles of three liver cancer cell lines with different metastatic potential through a microarray assay and focused on the candidate miR-3682-3p, which had lower expression in highly metastatic liver cancer cells than in poorly metastatic liver cancer cells. In terms of mechanism, we found that miR-3682-3p could suppress HUVEC angiogenesis, migration, and invasion by targeting ANGPT1 through the RAS-MEK1/2-ERK1/2 pathway. Our study illuminates a new molecular mechanism of angiogenesis and offers new potential therapeutic targets in tumor angiogenesis.

## Materials and Methods

### Cell Lines and Culture

Human umbilical vein endothelial cells (HUVECs) and the human liver cancer cell line PLC/RFP/5 were purchased from the Chinese Academy of Sciences Cell Bank (Shanghai, China). HUVECs were maintained in endothelial cell medium (ScienCell, United States). The HCCLM3 and MHCC97L cell lines, which were obtained from the Liver Cancer Institute of Zhongshan Hospital (Shanghai, China), as well as the PLC/RFP/5 cell line were cultured in DMEM supplemented with 10% fetal bovine serum (Gibco, United States). All cell lines were cultured in a humidified incubator containing 5% CO_2_ at 37°C.

### Extraction of Exosomes From Cell Culture Medium

All human liver cancer cell lines (HCCLM3, MHCC97L, and PLC/RFP/5) were plated in 10-cm plates and cultured to 70% confluence at equal numbers. The cells were then washed with serum-free DMEM twice and refreshed with 10 mL DMEM. The conditioned medium was harvested 48 h later and filtered through 0.22-μm filters (Millipore, United States). The supernatants were subjected to centrifugation for 30 min at 2,000 × *g* and 4°C and then for 20 min at 1,000 × *g* and 4°C to remove cell debris and ultracentrifuged at 110,000 × *g* and 4°C for 70 min to collect extracellular vesicles. The pellets were washed twice with 1 × PBS, ultracentrifuged at 110,000 × *g* for 70 min in a Beckman Optima L-100XP ultracentrifuge using a SW 32Ti rotor (Beckman Coulter, Germany), dissolved in 50–100 μl 1 × PBS and stored at −80°C.

### Transmission Electron Microscopy

Transmission electron microscopy (TEM) was performed to evaluate the morphology of isolated exosomes as previously described (Thery et al., 2006). Samples were fixed with 2% paraformaldehyde for 2 h at room temperature, loaded on film copper-mesh electron microscopy grids, and negatively stained with 2% uranyl acetate for 2 min. Images were captured with a Tecnai G2 transmission electron microscope (FEI Company, United States) operating at 120 kV.

### Nanoparticle Tracking Analysis

The size and number of isolated exosomes were assessed with a NanoSight NS 300 (NanoSight, Malvern, PA, United Kingdom) using NTA 3.3 software, as described elsewhere (Hazan-Halevy et al., 2015). In brief, samples were diluted with 1× PBS and measured at a concentration between 2 × 10^8^ and 7 × 10^8^ particles/ml in triplicate.

### Interactions Between HUVECs and Exosomes

Human umbilical vein endothelial cells were incubated in complete medium with exosomes pretreated with DiO (Beyotime, China) for 8 h at 37°C, washed twice with 1× PBS and then fixed in 4% formaldehyde for 10 min. Next, the cells were costained with DAPI (Thermo Fisher Scientific, United States) for 15 min and observed using a Zeiss SP2 confocal system (Leica, Germany).

### Western Blot Analysis

Exosomes and cells were lysed in RIPA buffer containing 1% protease inhibitor PMSF, and their protein concentrations were determined by the BCA protein assay (Beyotime, China). The same amounts of total lysates were separated by electrophoresis on 10% SDS-polyacrylamide gels at 80 V for 0.5 h followed by 120 V for 1 h and transferred to polyvinylidene difluoride (PVDF) membranes (Millipore, Billerica, MA, United States) at 300 mA for 1.5 h. The surface markers CD9 (ab92726, 1:500), CD81 (ab109201, 1:1000), Alix (ab186429, 1:1000), and TSG101 (ab125011, 1:1000) were used to identify exosomes. In addition, the following antibodies were used as the primary antibody: anti-RAS (#3339, CST, 1:1000), anti-MEK1/2 (ab178876, 1:2000), anti-p-MEK1/2 (Ser217/221) (#9154, CST, 1:1000), anti-ERK1/2 (Thr202/Tyr204) (#4370, CST, 1:2000), anti-p-ERK1/2 (#4695, CST, 1:1000), and anti-GAPDH (60004-1-Ig, Proteintech). Blots were detected by enhanced chemiluminescence after incubation with the secondary antibody.

### Microarray Analysis of Exosomal miRNAs

Total RNA was extracted from HCC-derived exosomes using TRIzol reagent (Invitrogen, United States), and exosomal miRNA microarray analysis was conducted using Six Agilent Human miRNA 8^∗^60K (Agilent Technologies, United States) at Wayen Corporation (Shanghai, China). Exosomal miRNAs were labeled and hybridized using the miRNA Complete Labeling and Hyb Kit (Agilent Technologies, United States) according to the manufacturer’s instructions. The microarray slides were scanned with an Agilent Microarray Scanner (Agilent Technologies, United States), and microarray images were analyzed using Agilent Feature Extraction software v10.7.

### Capillary Tube Formation Assay

For the capillary tube formation assay, 50 μl Matrigel (BD Matrigel 356234) was added to each well of 96-well plates and allowed to polymerize at 37°C for 30 min. Then, HUVECs were pretreated with 5 μg HCC-Exos for 24 h, resuspended in FBS-free medium and seeded at the bottom of each well at a density of 2 × 10^4^ cells/well. After culturing for 6 h, the HUVECs were examined for capillary-like structure formation using an inverted phase-contrast microscope. The branch points which represent the degree of angiogenesis *in vitro*, were scanned and counted in five random microscopic fields (100×) using ImageJ software.

### Cell Migration and Invasion Assays

Human umbilical vein endothelial cell migration and invasion were analyzed using transwell inserts (8-μm pore size; Corning) according to the manufacturer’s protocol. In brief, HUVECs were plated in the upper chamber, which was uncoated (1 × 10^5^ cells in 200 μl serum-free DMEM for the migration assay) or coated with 100 μl of Matrigel (BD Matrigel 356234) diluted 8× in DMEM (2 × 10^5^ cells in 200 μl serum-free DMEM for the invasion assay), and DMEM containing 10% FBS (500 μl) was added to the lower chamber as a chemoattractant. Subsequently, the HUVECs were treated with HCC-Exos or 1× PBS. After 24 h of incubation at 37°C and 5% CO_2_, the cells on the upper side of the membrane were removed with cotton swabs. Following this, the migrated or invaded cells on the underside of the membrane were fixed in 4% paraformaldehyde for 20 min at room temperature and then stained with 0.1% crystal violet for 10 min. Five random fields were imaged and counted. The experiments were performed in triplicate independently.

### *In vivo* Matrigel Plug Assay

To examine the roles of HCC-Exos in angiogenesis *in vivo*, 12 six-week-old BALB/c nude mice were injected subcutaneously with 500 μl Matrigel-reduced growth factor (BD Matrigel, 356230) containing HCCLM3, MHCC97L or PLC/RFP/5 Exos (100 μg) or 500 μl 1× PBS as a control. The mice were sacrificed, and the Matrigel plugs were harvested on day 22 after initial injection. All animal experimental procedures were conducted under guidelines approved by the Animal Care Committee of Fudan University (Shanghai, China).

### Histology

Matrigel plugs were fixed in 4% formaldehyde, embedded in paraffin, cut transversely into blocks and then sectioned at 5-μm thickness. Paraffin sections of Matrigel plugs were stained with hematoxylin and eosin (H&E). Sections were subjected to rehydration and antigen retrieval, blocked in 5% BSA and then incubated with a primary antibody against CD31 (ab182981) overnight, followed by incubation with a secondary antibody for 30 min and staining with DAB peroxidase substrate (Maxim, #DAB4033). Cells with brown granules in the cytomembrane were deemed positive staining cells.

### Lentivirus and RNA Oligonucleotides

A miR-3682-3p mimic and mimic negative control were purchased from Genomeditech (Shanghai, China) and transfected into HUVECs using Lipofectamine 2000 reagent (Invitrogen, United States). In addition, to overexpress ANGPT1, lentiviral vectors encoding ANGPT1 were constructed using the GV492 vector (GeneChem Inc., Shanghai, China) based on the GenBank information for ANGPT1 (NM_001146). The empty vector (LV-vector) was used as a negative control. HUVECs were transfected with a lentivirus to establish a stable ANGPT1-overexpressing cell line using polybrene and subsequently selected with puromycin for 1 week following the instructions of the manufacturer.

### miR-3682-3p Target Prediction

We used the TargetScan database^1^ to predict and analyze the target genes of miRNA-3682-3p, and predicted binding sites were found to be at positions 852-859 and 2321-2328 in the 3′-UTR of the ANGPT1 mRNA transcript.

### Luciferase Reporter Assay

For luciferase activity assays, HEK293T cells were seeded in a 96-well plate and cultured to 70% confluence. The cells were cotransfected with miR-3682-3p or NC duplex, pRL-CMV (expressing Renilla luciferase) and a firefly luciferase activity plasmid that contained either a mutant 3′-UTR of the target gene or the wild-type 3′-UTR using Lipofectamine 2000 reagent (Invitrogen, United States). Luciferase activity was detected 48 h after transfection using a dual-luciferase reporter assay kit (Promega, United States).

### Statistical Analysis

Statistical analyses were performed using GraphPad Prism 5 (GraphPad, United States). Each experiment was performed at least in triplicate, and all data are presented as the mean ± standard error. Two groups were compared by Student’s *t*-test, and multiple groups were analyzed using one-way ANOVA.*p* < 0.05 was considered significant.

## Results

### Isolation and Identification of HCC-Derived Exosomes

**FIGURE 1:**
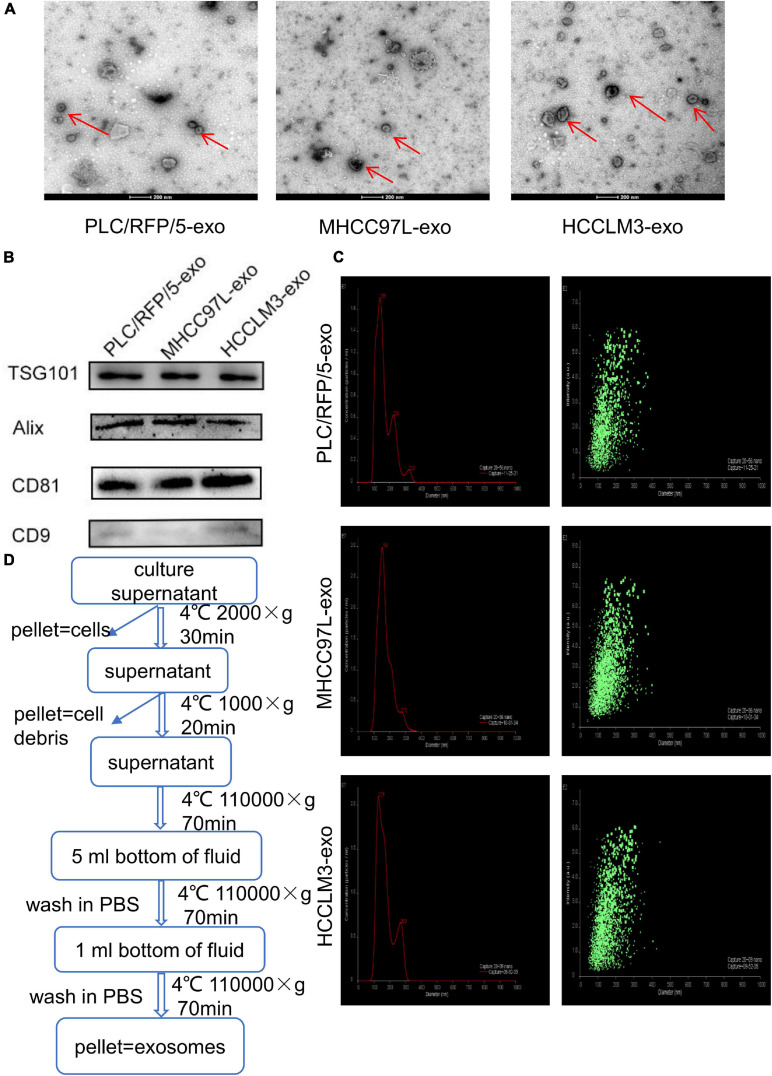
Characterization of exosomes derived from liver cancer cells. **(A)** Transmission electron microscopy analysis of exosomes (Exos) derived from HCC cell lines (HCCLM3, MHCC97L, and PLC/RFP/5). Scale bar, 200 nm. **(B)** Immunoblotting assay of exosomal biomarkers in different liver cancer cells. **(C)** Mean diameter and size distribution of HCC-Exos was detected by Nanoparticle tracking analysis. **(D)** Strategy for exosomes purifcation from the HCC cell lines culture supernatants based on differential centrifugations.

Exosomes were isolated from the culture supernatants of the HCC cell lines HCCLM3, MHCC97L, and PLC/RFP/5 by the differential ultracentrifugation method, as shown in Figure 1D. TEM was used to observe the morphology and structure of isolated particles, which exhibited a round or cup-shaped morphology (Figure 1A). The characteristic exosomal markers TSG101, Alix, CD9, and CD81 were detected by western blotting (Figure 1B). Nanoparticle tracking analysis (NTA) was performed to analyze the size and distribution of particles obtained from HCCLM3, MHCC97L, and PLC/RFP/5 cells, and the results indicated that the mean diameter of the derived particles were 129, 154, and 139 nm, respectively (Figure 1C). The above results indicated that the particles we isolated were exosomes.

### HCC-Derived Exosomes Were Internalized by Endothelial Cells

**FIGURE 2:**
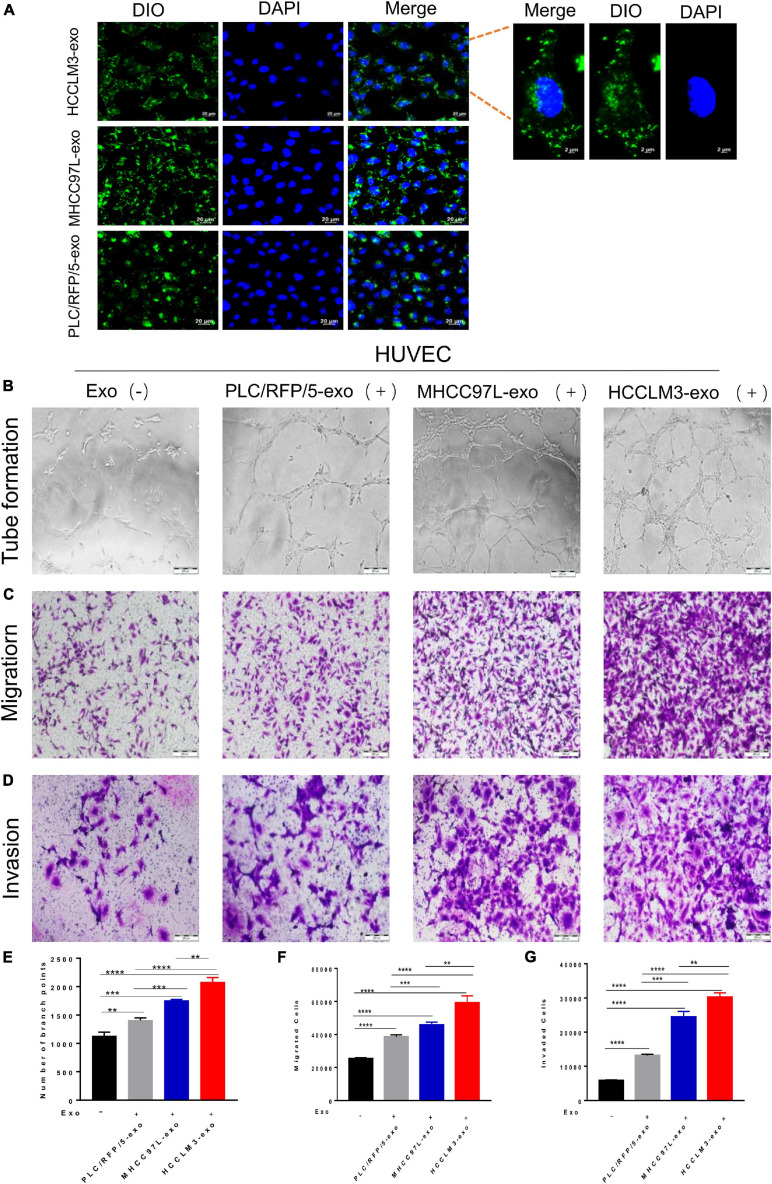
HCC exosomes promoted HUVECs tube formation, migration, and invasion *in vitro* and this effect increases with increasing metastatic potential of HCC cells. **(A)** Confocal imaging showed the delivery of Dio-labeled exosomes (green) to DAPI-labeled HUVECs (blue) and representative images were presented. Scale bar, 20 μm. **(B–D)** Tube formation assays **(B)**, migration assays **(C)**, and invasion assays **(D)** of HUVECs treated with equal quantities of exosomes derived from different liver cancer cells or blank control. Scale bar, 200 μm. **(E–G)** Each experiment was performed three times independently and results were analyzed with ImageJ software. (*p < 0.05; **p < 0.01; ***p < 0.001; ****p < 0.0001).

To explore whether HCC-secreted exosomes can interact with endothelial cells, HCC-Exos were fluorescently labeled with DiO (Beyotime, Shanghai, China) and then incubated with HUVECs. After a 6-h incubation, the HUVECs were labeled with DAPI (Thermo Fisher Scientific, United States) and captured using a confocal microscope. The images showed that DiO-labeled HCC-Exos were internalized into the cytoplasm of HUVECs, indicating that HCC-Exos could be absorbed by endothelial cells (Figure 2A).

### HCC-Derived Exosomes Promote Endothelial Cell Tube Formation, Migration, and Invasion *in vitro*

Many studies have indicated that tumor-derived exosomes can act as cargo carriers to transport signals regulating the tumor microenvironment. To explore the biological functions of HCC-derived exosomes in tumor angiogenesis, a hallmark of invasive tumor growth and metastasis, HUVECs were incubated with different kinds of collected HCC-Exos at equal amounts. In a capillary tube formation assay, the number of nodes formed by HUVECs incubated with exosomes obtained from HCCLM3, MHCC97L, or PLC/RFP/5 cells were significantly increased compared to those of control-treated HUVECs, which were incubated with an equal volume of 1× PBS (Figures 2B,E). A transwell migration assay and subsequent manual counting indicated that the number of migrated HUVECs significantly increased when the cells were incubated with HCC-secreted exosomes (Figure 2C), as shown in Figure 2F. Meanwhile, the number of invaded control cells was dramatically decreased, as measured by the transwell invasion assays (Figures 2D,G). Hence, we concluded that exosomes purified from human liver cancer lines promote endothelial cell tube formation, migration, and invasion. In addition, we also found that this effect increases with increasing metastatic potential for HCC cells.

### HCC-Derived Exosomes Promote Angiogenesis *in vivo*

**FIGURE 3:**
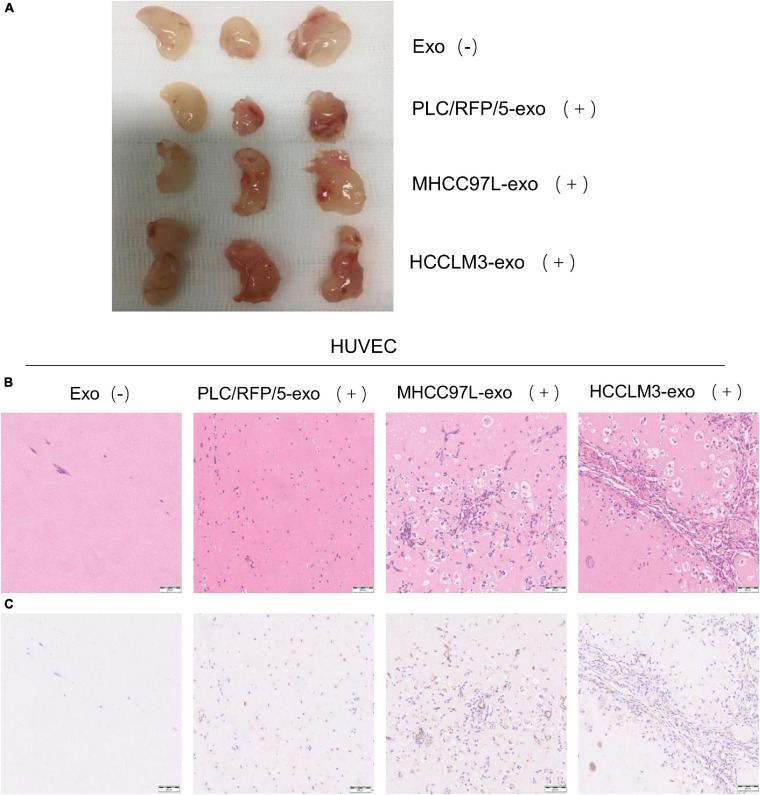
HCC derived exosomes promote angiogenesis *in vivo*. **(A)** Matrigel plug assay performed by subcutaneous injection of Matrigel mixed with/without HCC exosomes (100 μg) in 12 BALB/c nude mice (*n* = 3/group). **(B)** Representative of H&E staining of matrigel plug: PBS; PLC/RFP/5 EXOs; MHCC97L EXOs; and HCCLM3 EXOs. (×100), scale bar, 200 μm. **(C)** Matrigel plug were formalin-fixed, paraffin-embedded, sliced and stained with CD31 and representative images of each group were presented.

To further evaluate the effects of HCC-derived exosomes on proangiogenic function *in vivo*, Matrigel containing 100 μg exosomes derived from HCCLM3, MHCC97L, or PLC/RFP5 cells or an equivalent amount of 1× PBS as a control was injected subcutaneously into BALB/c nude mice (*n* = 3 per group). The implanted Matrigel plugs were harvested 21 days after injection (Figure 3A). Formaldehyde-fixed, paraffin-embedded Matrigel plug sections were subjected to histological examination (H&E staining), and the results indicated that there were more endothelial cells and blood vessels in the HCC-Exo groups than in the control group (Figure 3B). Immunohistochemistry was conducted to determine the effect of HCC-Exos on angiogenesis by staining for the vascular marker CD31. The HCC-Exo groups showed significantly higher capillary density than the control group (Figure 3C), indicating a potential role for HCC-Exos in angiogenesis. Moreover, the exosomes derived from HCCLM3/MHCC97L cells, which are highly invasive and have a metastatic potential, had a more significant effect on angiogenesis *in vivo* than those derived from PLC/RFP/5 cells, which are poorly invasive and have a low metastatic potential, which was consistent with the results of the *in vitro* experiments. The *in vivo* Matrigel plug assays revealed that HCC-Exos promote angiogenesis and that this effect becomes more obvious as the invasiveness and metastatic potential of the source HCC cells increase.

### Exosomal miR-3682-3p Attenuated Endothelial Cell Tube Formation, Migration, and Invasion

**FIGURE 4:**
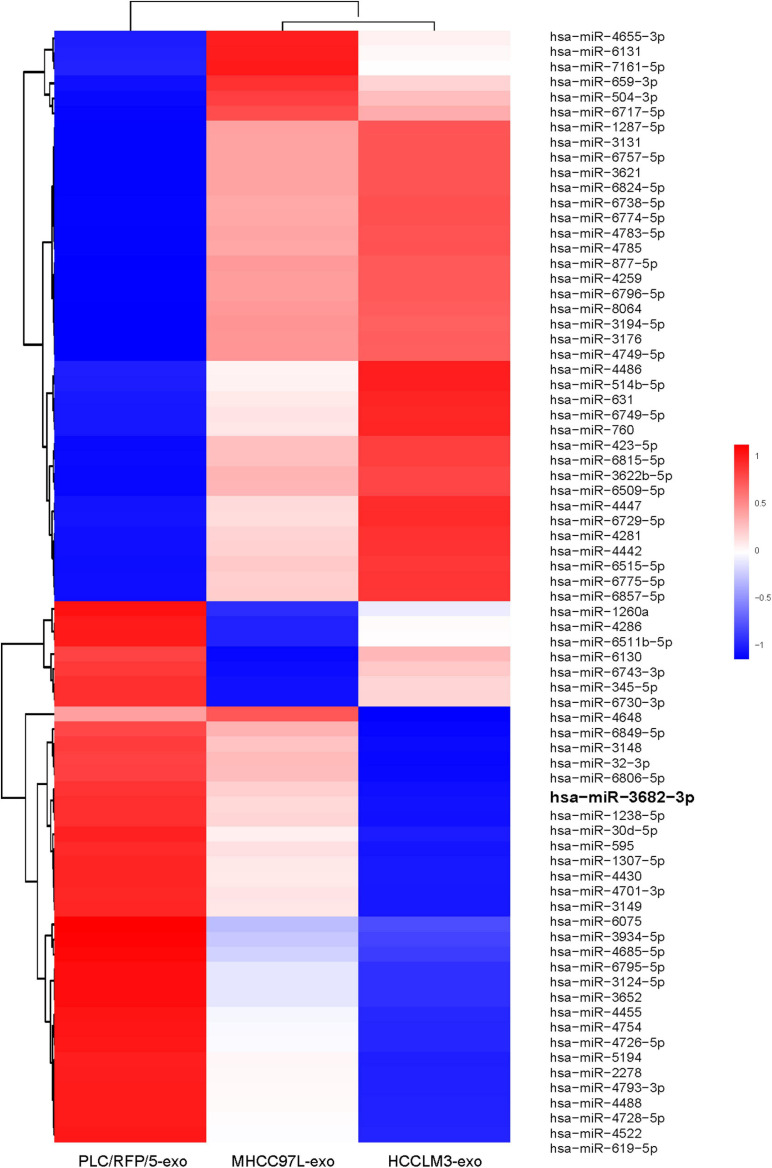
Microarray analysis of exosomal miRNAs from different liver cancer cells were presented in a heatmap.

**FIGURE 5:**
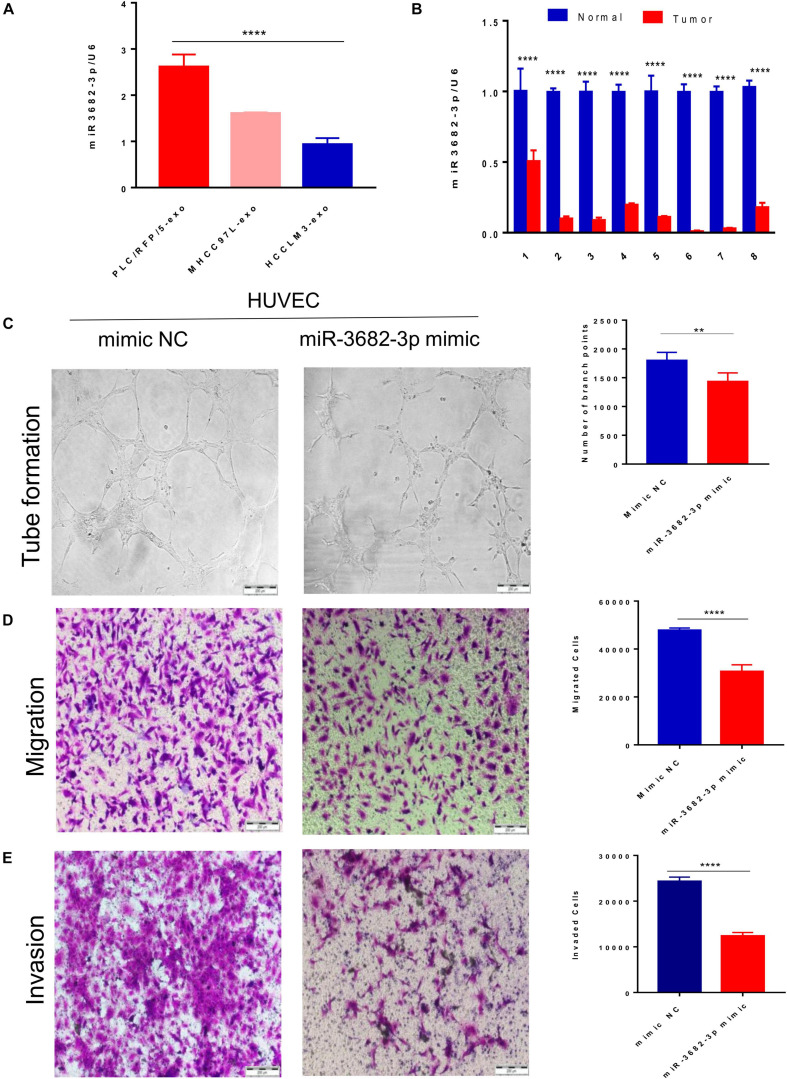
Exosomal miR-3682-3p secreted by liver cancer cells suppressed HUVECs angiogenesis. **(A)** The microarray results were verified by qPCR. **(B)** The miR-3682-3p expressions in 8 frozen HCC tissues were generally decreased as compared with their corresponding normal tumor–adjacent tissues. **(C–E)** Tube formation assays **(C)**, migration assays **(D)**, and invasion assays **(E)** of HUVECs transfected with miR-3682-3p-mimic or negative control. Scale bar, 200 μm. (*p < 0.05; **i < 0.01; ***p < 0.001; ****p < 0.0001.)

We next explored how HCC-derived exosomes promote angiogenesis *in vitro* and *in vivo*. To identify the proangiogenic components in exosomes, we performed miRNA microarray analysis of HCC-Exos because previous studies indicated that miRNAs are abundant in exosomes and have significant roles in cell–cell communication (Valadi et al., 2007). The differentially expressed miRNAs were compared and are shown in a heat map (Figure 4). In this study, a fold change >2 and *p*-value < 0.001 were applied to select proangiogenic miRNAs. MiR-3682-3p was chosen for further analysis because the expression of miR-3682-3p significantly decreased as the invasiveness and metastatic potential of HCC cell lines increased and because this miRNA has not been implicated in the suppression of angiogenesis. We verified the microarray results by qPCR (Figure 5A), and we also found that miR-3682-3p expression in 8 frozen HCC tissue sections was generally decreased compared with that in corresponding tumor-adjacent normal tissue samples (Figure 5B).

Human umbilical vein endothelial cells were infected with a miR-3682-3p mimic or negative control to explore the role of exosomal miR-3682-3p in angiogenesis. First, we found that miR-3682-3p inhibited endothelial cell tube formation compared with the negative control by performing capillary tube formation assays with HUVECs (Figure 5C). Second, migration assays were performed, and the results showed that fewer HUVECs migrated in the miR-3682-3p mimic group than in the negative control group (Figure 5D). Additionally, Transwell invasion assays confirmed that increased expression of miR-3682-3p could remarkably attenuate the invasive ability of HUVECs (Figure 5E). Quantification of the results of the tube formation assays, migration assays, and invasion assays are also shown in this figure. These data indicate that exosomal miR-3682-3p attenuates the tube formation, migration, and invasion of endothelial cells.

### Exosomal miR-3682-3p Attenuated Endothelial Cell Tube Formation, Migration, and Invasion by Targeting ANGPT1

**FIGURE 6:**
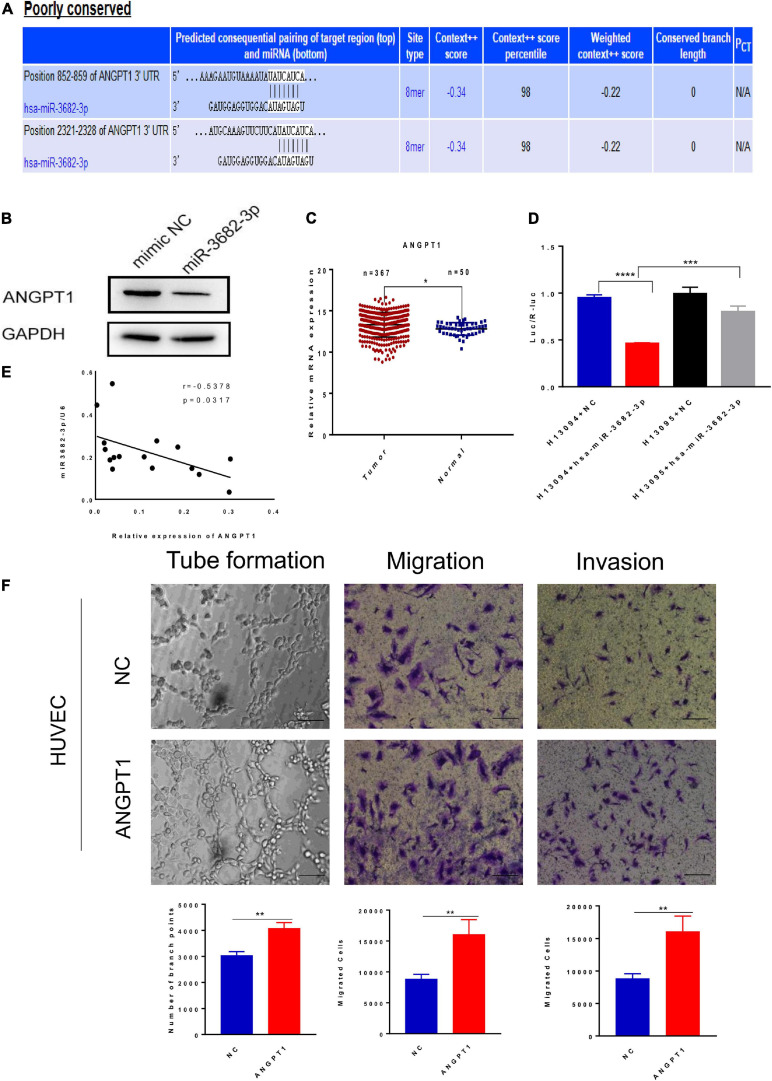
Exosomal miR-3682-3p downregulated the expression of ANGPT1. **(A)** There were two potential binding sites for miR-3682-3p in the ANGPT1 transcript within its 3′–UTR (position 852-859 and 2321-2328). **(B)** The protein levels of ANGPT1 were determined by western blot. **(C)** ANGPT1 mRNA expression in hepatocellular carcinoma patients from the TCGA database. **(D)** miR–3682–3p significantly suppressed the luciferase activity of the ANGPT1–WT but had no obvious effects on the luciferase activity of ANGPT1-MUT. **(E)** Negative correlation between miR-3682-3p and ANGPT1 mRNA in 16 HCC tissues. **(F)** Tube formation assays, migration assays, and invasion assays of HUVECs transfected with ANGPT1-OE lentivirus or negative control. Scale bar, 200 μm. (*p < 0.05; **p < 0.01; ***p < 0.001; ****p < 0.0001).

MicroRNAs have emerged as important regulators of diverse biological processes, but the regulatory mechanism of HCC exosomal miRNA-mediated angiogenesis remains unclear. We next explored how HCC-secreted miR-3682-3p attenuate the tube formation, migration, and invasion of endothelial cells. MicroRNAs, as a category of endogenous non-coding RNAs with a length of 22–24 nucleotides, can regulate target gene expression at the posttranscriptional level. The putative targets of miR-3682-3p were predicted using the TargetScan databases, which showed that there are two potential binding sites for miR-3682-3p in the ANGPT1 transcript (Figure 6A). For this reason, we wondered whether ANGPT1, which has not been reported as a target gene of miR-3682-3p, affects angiogenesis, and we performed further research to address this question. Luciferase reporter assays were conducted to verify whether the ANGPT1 gene is a direct target gene of miR-3682-3p. The 3′-UTR of the ANGPT1 transcript sequence containing the predicted miR-3682-3p binding sites was cloned into a plasmid vector, and the plasmid vector was transfected into HEK293T cells. Luciferase activity results revealed that cotransfection of miR-3682-3p significantly inhibited the activity of the firefly luciferase reporter carrying the wild-type 3′-UTR of the ANGPT1 transcript, whereas this effect was abrogated when the predicted binding sites in the 3′-UTR were mutated (Figure 6D). Immunofluorescence staining confirmed that ANGPT1 gene expression was markedly downregulated after HUVECs were transfected with the miR-3682-3p mimic compared with the negative control (Figure 6B). In addition, ANGPT1 mRNA expression in HCC patients from the TCGA database was analyzed, and we found that this expression was generally increased in tumor tissues compared with tumor-adjacent normal tissues (Figure 6C). To investigate the relationship of miR-3682-3p and ANGPT1 in HCC patients, total RNAs were first extracted from 16 HCC patients randomly and then the level of miR-3682-3p and ANGPT1 was analyzed by RT-PCR. There existed a significant negative correlation between miR-3682-3p and ANGPT1 in HCC tissues, which was in line previous observations (*r* = −0.5378, *p* = 0.0317; Figure 6E). Tube formation, migration, and invasion assays were conducted, and the results indicated that ANGPT1 enhanced the tube formation, migration, and invasion of endothelial cells *in vitro* (Figure 6F). Overall, these data revealed that exosomal miR-3682-3p secreted from HCC cells could inhibit tube formation, invasion and migration by targeting ANGPT1 in endothelial cells.

### Exosomal miR-3682-3p Suppresses ANGPT1 Expression via RAS-MEK1/2-ERK1/2 Signaling

**FIGURE 7:**
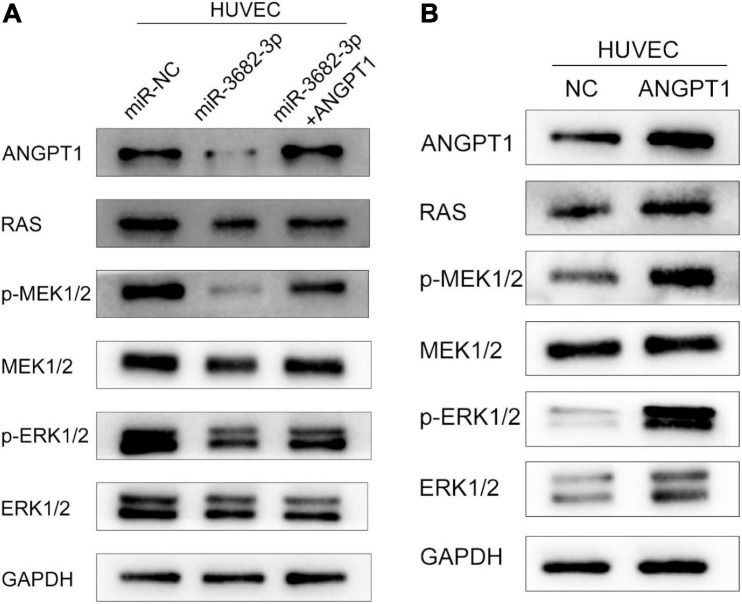
The hepatocellular carcinoma exosomal-miR-3682-3p suppressed HUVECs via ANGPT1-RAS-MEK1/2-ERK1/2 signaling axis. **(A)** Exosomal-miR-3682-3p suppressd the expression of p-MEK1/2, p-ERK1/2, and ANGPT1 and this effet can be counteract by ANGPT1. **(B)** Western blot assays showed that overexpression of ANGPT1 also activated the RAS-MEK1/2-ERK1/2 pathway in HUVECs.

The RAS-MEK1/2-ERK1/2 pathway is a pivotal intracellular signaling pathway that regulates many biological functions and cellular processes, including proliferation, migration, invasion, differentiation, apoptosis, and metabolism (Xu et al., 2017). Hyperactivation of the RAS-MEK1/2-ERK1/2 signaling pathway is regarded as a hallmark driving angiogenesis and tumorigenesis in various human cancers (Peng and Gassama-Diagne, 2017). In view of this, we examined whether miR-3682-3p and ANGPT1 modulation affect three endogenous targets of RAS-MEK1/2-ERK1/2 signaling in HUVECs. We found that pathway activation was suppressed by the miR-3682-3p mimic, as demonstrated by significant reductions in the levels of the activated forms of RAS, p-MEK1/2, and p-ERK1/2, and this effect could be rescued by co-overexpression of ANGPT1. In addition, overexpression of ANGPT1 also activated the RAS-MEK1/2-ERK1/2 pathway in HUVECs. These findings imply that miR-3682-3p may suppress angiogenesis by repressing ANGPT1 gene expression via RAS-MEK1/2-ERK1/2 signaling in HUVECs (Figures 7A,B).

## Discussion

Accumulating evidence indicates that exosomes play important roles in cell–cell communication under physiological and pathophysiological conditions (Sahoo and Losordo, 2014; Sluijter et al., 2014). Multiple stromal cells, including endothelial cells, fibroblasts, and macrophages, which contribute to the generation of metastatic tumor microenvironments, can be influenced by the horizontal transfer of functional molecules, such as miRNAs and other bioactive molecules in exosomes (Skog et al., 2008; Joyce and Pollard, 2009; Lima et al., 2009). Fang et al. (2018b) demonstrated that HCC cell-derived exosomes could deliver miR-1247-3p into fibroblasts and thereby convert normal fibroblasts into cancer-associated fibroblasts to promote the lung metastasis of liver cancer. Data from Liu et al. (2019) clearly showed that exosomal miR-192-5p played a significant role in inducing macrophage polarization toward a proinflammatory phenotype and activating hepatic inflammation. Wang et al. (2018) revealed that tumor-derived exosomes affected the PD1^+^ macrophage population in human gastric cancer. However, there are few studies addressing the mechanism involving exosomes in tumor angiogenesis. Thus, it is necessary to study the interaction between tumors and endothelial cells mediated by exosomes.

In our study, we found that exosomes purified from HCC cell lines (HCCLM3, MHCC97L, and PLC/RFP/5) could be delivered into endothelial cells and subsequently accelerate endothelial cell tube formation, migration, and invasion. *In vivo* Matrigel plug assays showed that more vessels were detected in the HCC-Exos groups than in the control group by analysis of Matrigel plug sections through immunohistochemical staining for the vascular marker CD31 and H&E staining. Furthermore, this effect was enhanced with increases in the invasiveness and metastatic potential of HCC cells. It is implied that HCC-secreted exosomes play an important role in angiogenesis.

To determine the functional contents and molecular mechanisms of HCC-derived exosomes, we analyzed the miRNA profiles of exosomes isolated from three different HCC cell lines using microarray analysis. We found that miR-3682-3p expression in poorly metastatic cell lines was significantly higher than that in highly metastatic cell lines. Additionally, miR-3682-3p expression in 8 frozen HCC tissue samples was generally lower than that in corresponding tumor-adjacent normal tissue samples. Then, we performed a series of functional experiments *in vitro* to assess the role of miR-3682-3p in tumor angiogenesis. The results showed that miR-3682-3p significantly suppressed tube formation, migration, and invasion of endothelial cells. Many previous studies have reported that miRNAs participate in the promotion of angiogenesis by regulating the expression of tumor promoters. For instance, Zeng et al. (2018) revealed that exosomal miR-25-3p regulated the expression of VEGFR2, ZO-1, occludin, and Claudin5 in endothelial cells by targeting KLF2 and KLF4, consequently promoting vascular permeability and angiogenesis. Lin et al. (2018) reported that exosomal miR-210 secreted from HCC cells could be delivered into endothelial cells and then directly inhibit the expression of SMAD4 and STAT6, resulting in enhanced angiogenesis. In addition, Fang et al. (2018a) found that exosomal miR-103 increased vascular permeability and promoted metastasis by targeting the junction protein p120 in HCC. In our study, we used the TargetScan database to predict the downstream regulators of miR-3682-3p and observed that the 3′-UTR of the ANGPT1 transcript exhibited two putative binding sites for miR-3682-3p at positions 852-859 and 2321-2328. Through a dual-luciferase assay and gain-of-function analysis, ANGPT1 was identified to be the direct target gene of miR-3682-3p, and ANGPT1 expression levels could be negatively regulated by miR-3682-3p. Functional experiments further revealed that ANGPT1 could enhance the tube formation, migration, and invasion of endothelial cells *in vitro*. In addition, Suri et al. (1998) showed that transgenic overexpression of angiopoietin-1 in the skin of mice produced larger, more numerous, and more highly branched vessels. That study corroborates our findings; thus, we hypothesized that miR-3682-3p suppresses angiogenesis by inhibiting the expression of ANGPT1.

Most proangiogenic factors activate the RAS-MEK1/2-ERK1/2 cascade, a signaling pathway that promotes angiogenesis by triggering the proliferation, migration, and invasion of endothelial cells (Mavria et al., 2006). MEK1/2-ERK1/2 inhibition is a prospective therapeutic approach for preventing angiogenesis and tumor growth and metastasis (Gourlaouen et al., 2013). We wondered whether miR-3682-3p performs its biological function by targeting ANGPT1 through the RAS-MEK1/2-ERK1/2 signaling pathway. Our experiments confirmed that miR-3682-3p inhibited the pathway by suppressing the expression of ANGPT1, RAS, p-MEK1/2, and p-ERK1/2. Moreover, we also found that ANGPT1 activated the RAS-MEK1/2-ERK1/2 pathway by western blot assays.

## Conclusion

In conclusion, the present study suggests that HCC-derived exosomal miR-3682-3p suppresses the expression of ANGPT1 and inhibits the RAS-MEK1/2-ERK1/2 signaling pathway in endothelial cells during angiogenesis. Our findings elucidate a new molecular mechanism underlying the crosstalk between HCC cells and endothelial cells mediated by exosomes. These new findings may provide insights for developing efficient therapeutic strategies to treat HCC by focusing on tumor angiogenesis.

## Standard Biosecurity and Institutional Safety Procedures Statement

We adhered to standard biosecurity and institutional safety procedures of Fudan University.

## Data Availability Statement

The original contributions presented in the study are included in the article/Supplementary Material, further inquiries can be directed to the corresponding author/s.

## Ethics Statement

The animal study was reviewed and approved by the Animal Care Committee of Fudan University (Shanghai, China).

## Author Contributions

B-BL contributed the study concept and critical design of this study. S-SD, D-DD, Z-FY, G-QZ, D-MG, JC, and YZ conducted the cell experiments. S-SD and D-DD analyzed and interpreted the data. B-BL, S-SD, and D-DD fulfill the initial manuscript. B-BL critically reviewed and revised the final manuscript. All authors contributed to the article and approved the submitted version.

## Conflict of Interest

The authors declare that the research was conducted in the absence of any commercial or financial relationships that could be construed as a potential conflict of interest.

## Footnotes

1 http://www.targetscan.org/vert_72/
