# Supplementary figures and images for: Exosomal miR-3682-3p Suppresses Angiogenesis by Targeting ANGPT1 via the RAS-MEK1/2-ERK1/2 Pathway in Hepatocellular Carcinoma
Source: Front Cell Dev Biol. 2021 Mar 29;9:633358. doi: 10.3389/fcell.2021.633358 (PMC8044774; doi:10.3389/fcell.2021.633358)

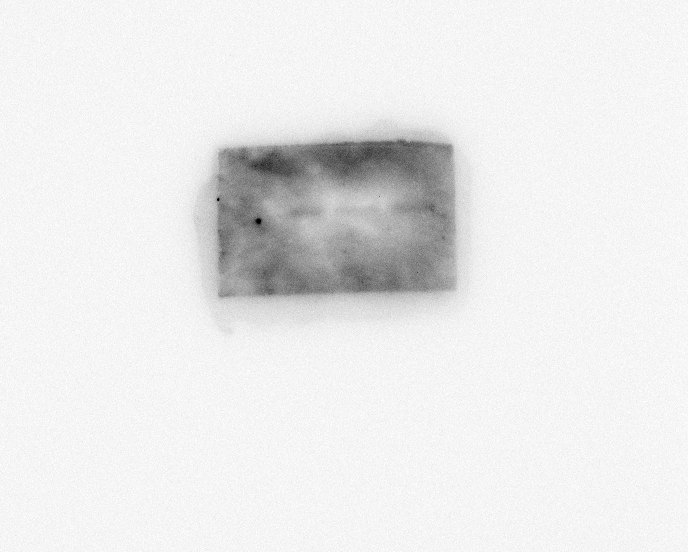

Supplement: Supplementary file 1 [file Data_Sheet_1.ZIP › Fig1-CD9.tif]

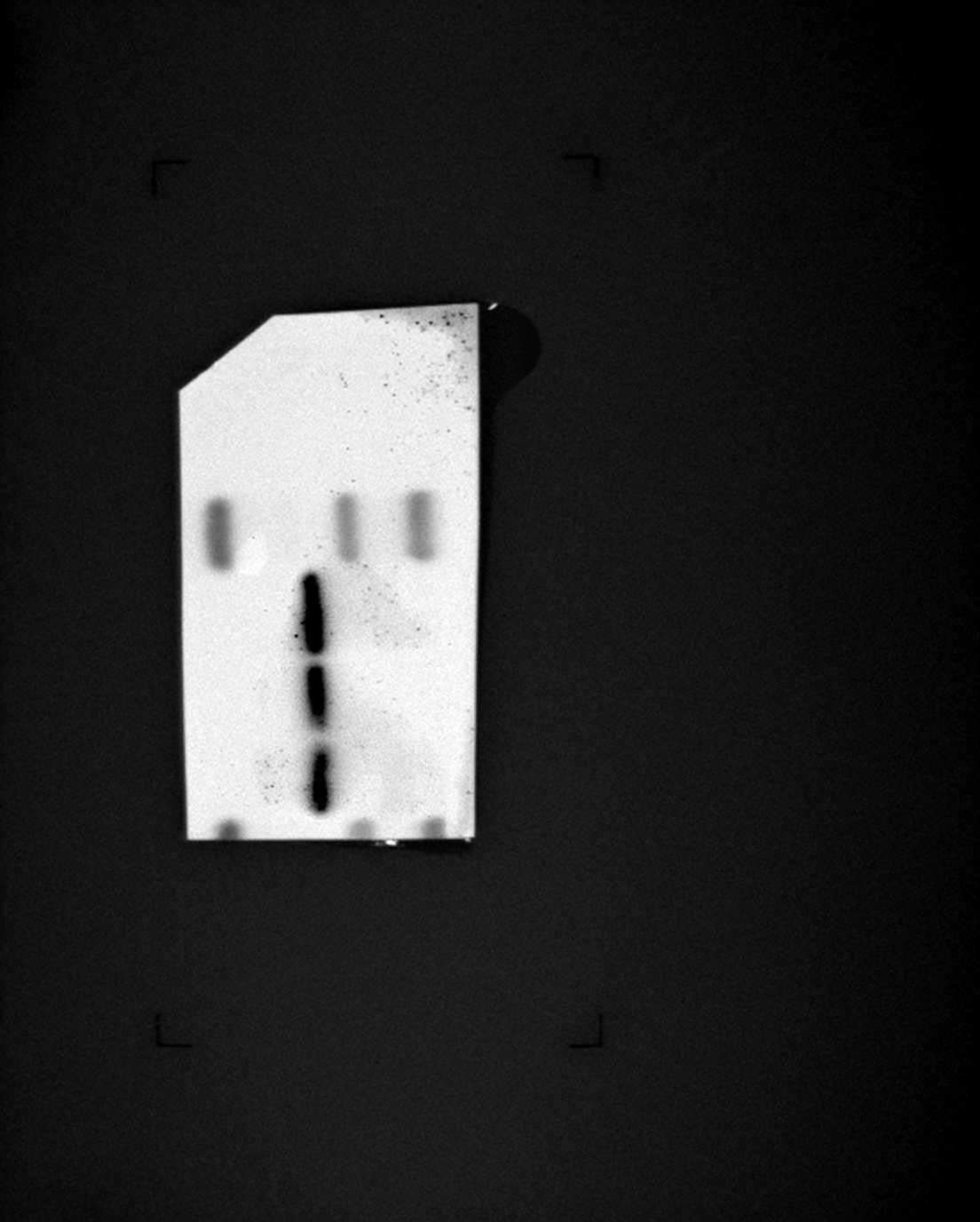

Supplement: Supplementary file 1 [file Data_Sheet_1.ZIP › Fig1-CD81.tif]

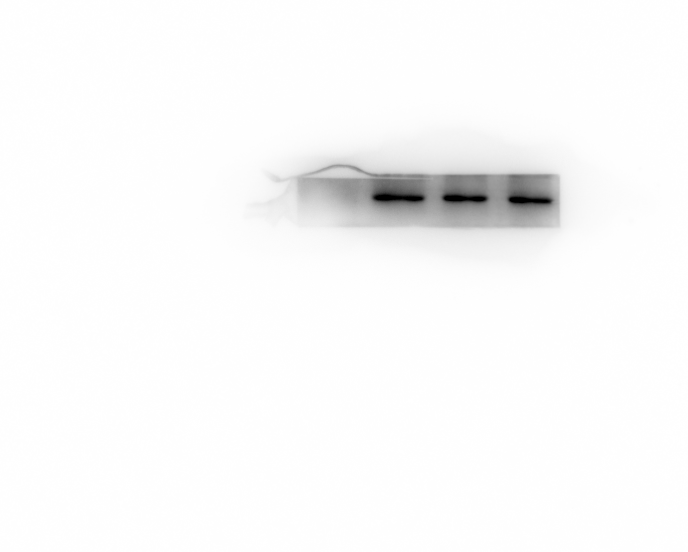

Supplement: Supplementary file 1 [file Data_Sheet_1.ZIP › Fig1-TSG101.tif]

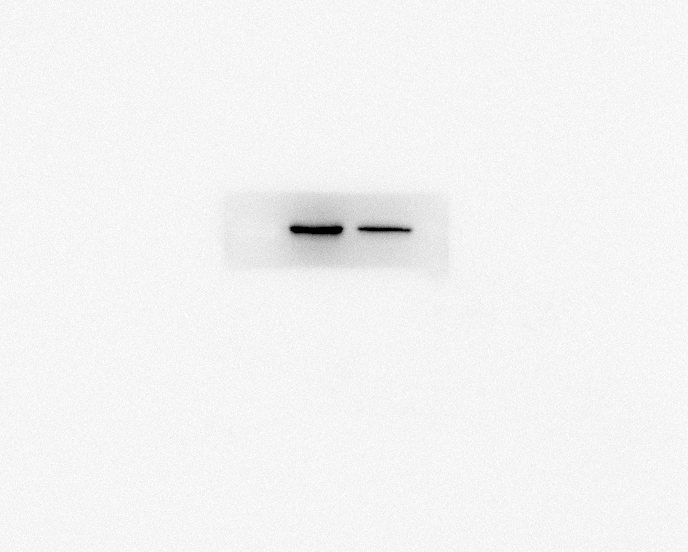

Supplement: Supplementary file 1 [file Data_Sheet_1.ZIP › Fig5-ANGPT1.tif]

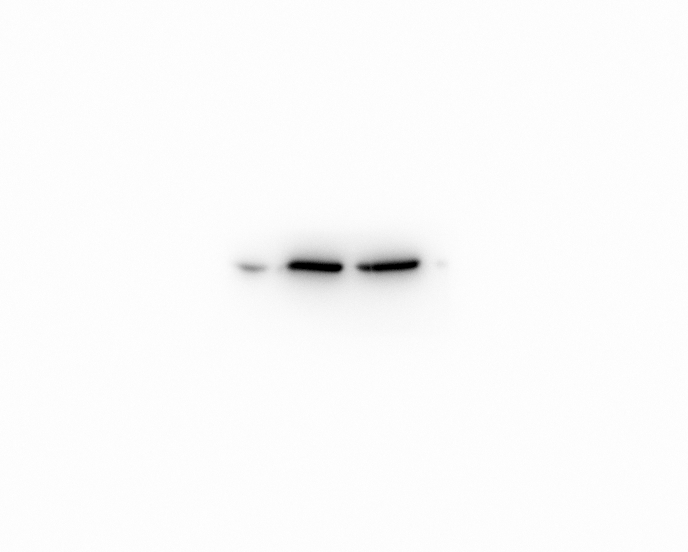

Supplement: Supplementary file 1 [file Data_Sheet_1.ZIP › Fig5-GAPDH.tif]

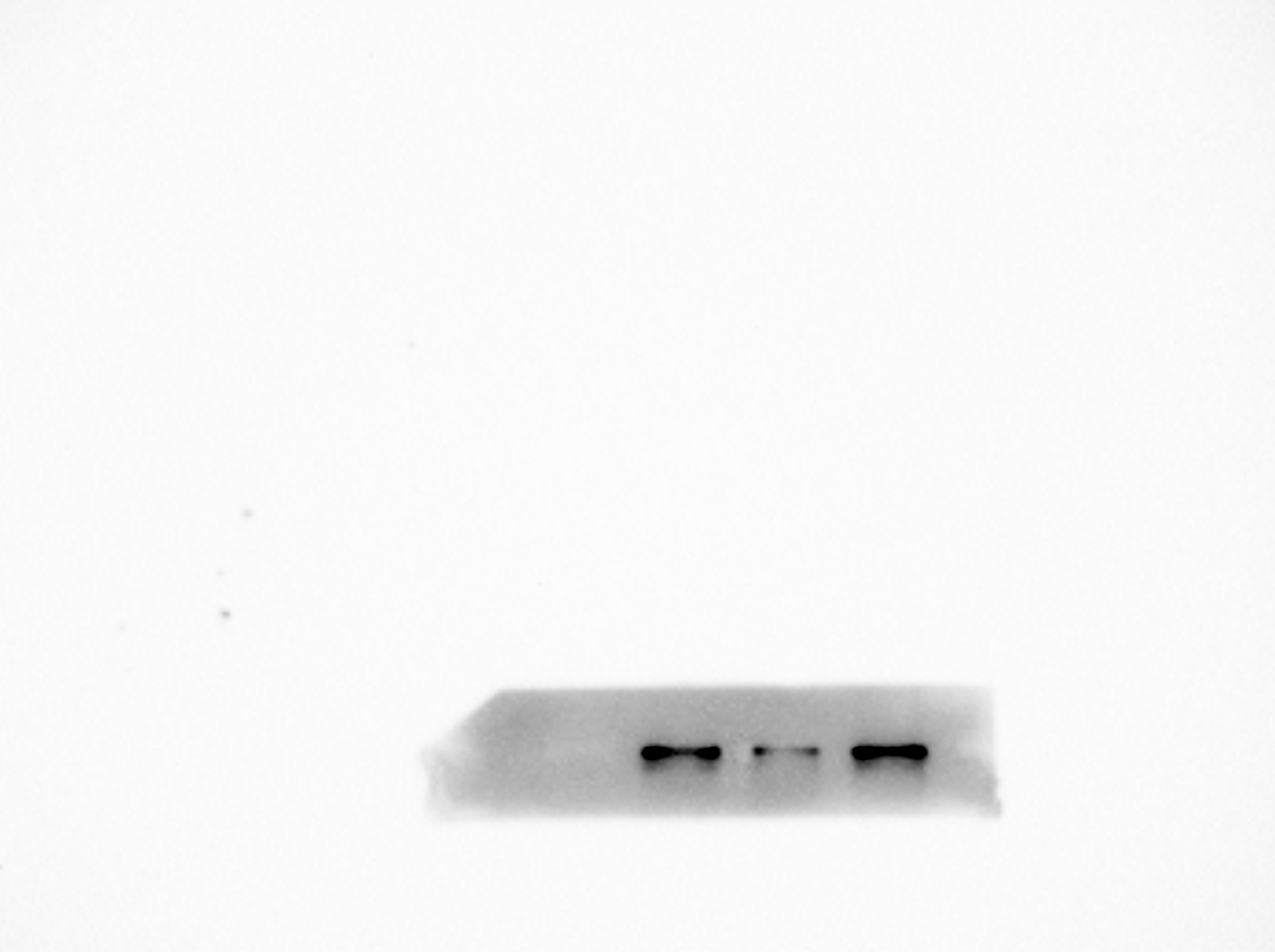

Supplement: Supplementary file 1 [file Data_Sheet_1.ZIP › Fig6A-ANGPT1.tif]

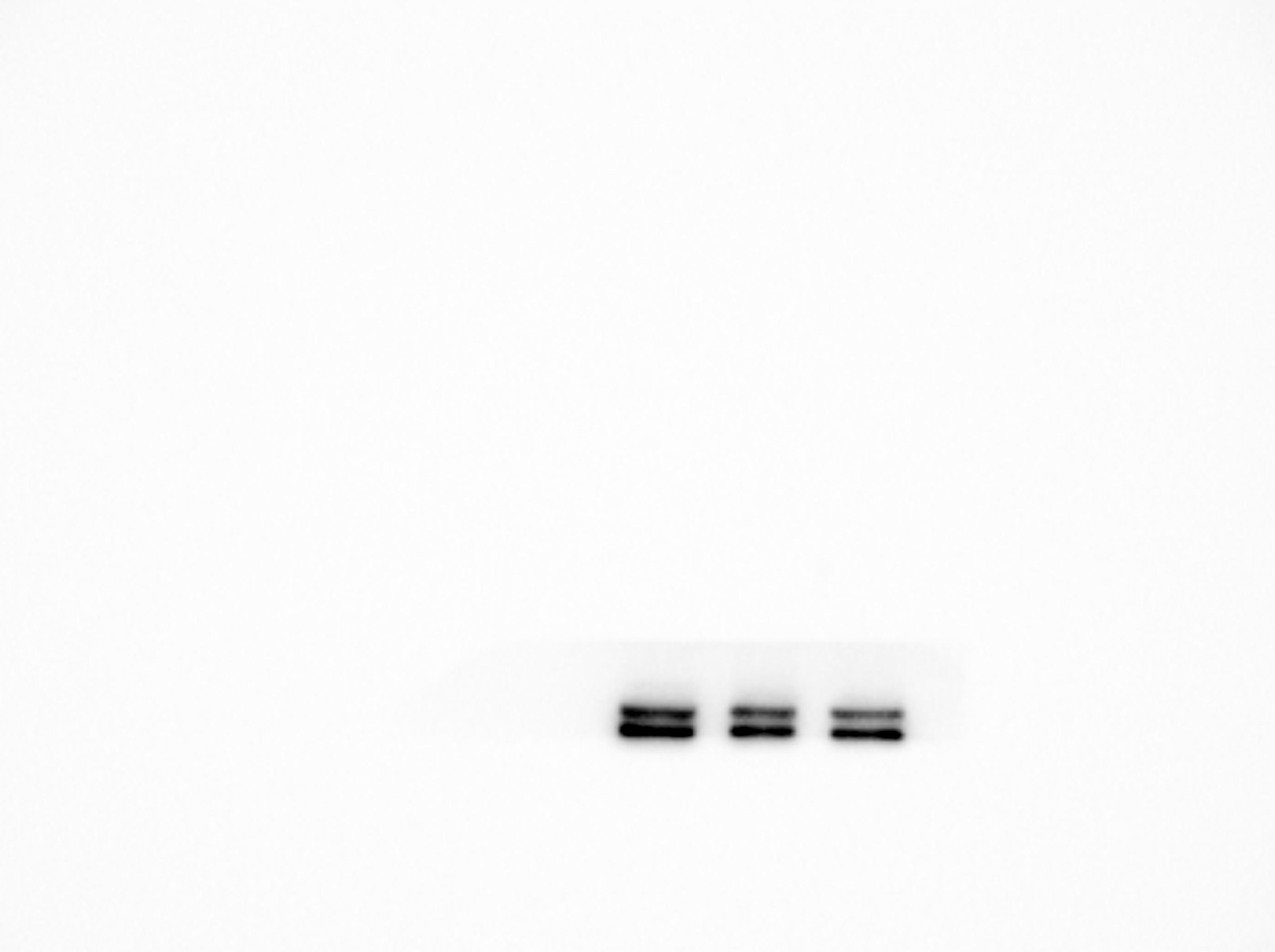

Supplement: Supplementary file 1 [file Data_Sheet_1.ZIP › Fig6A-ERK.tif]

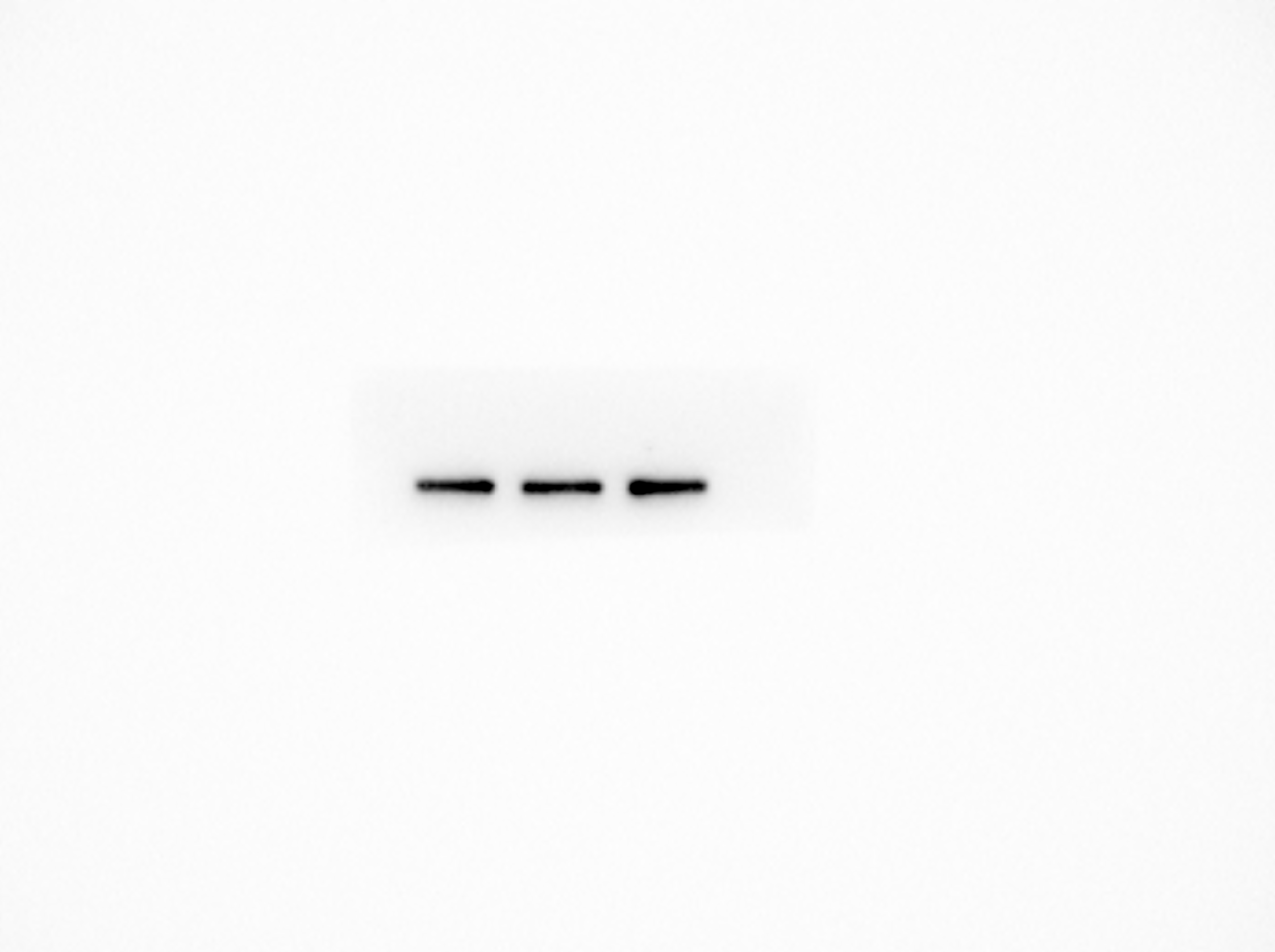

Supplement: Supplementary file 1 [file Data_Sheet_1.ZIP › Fig6A-GAPDH.tif]

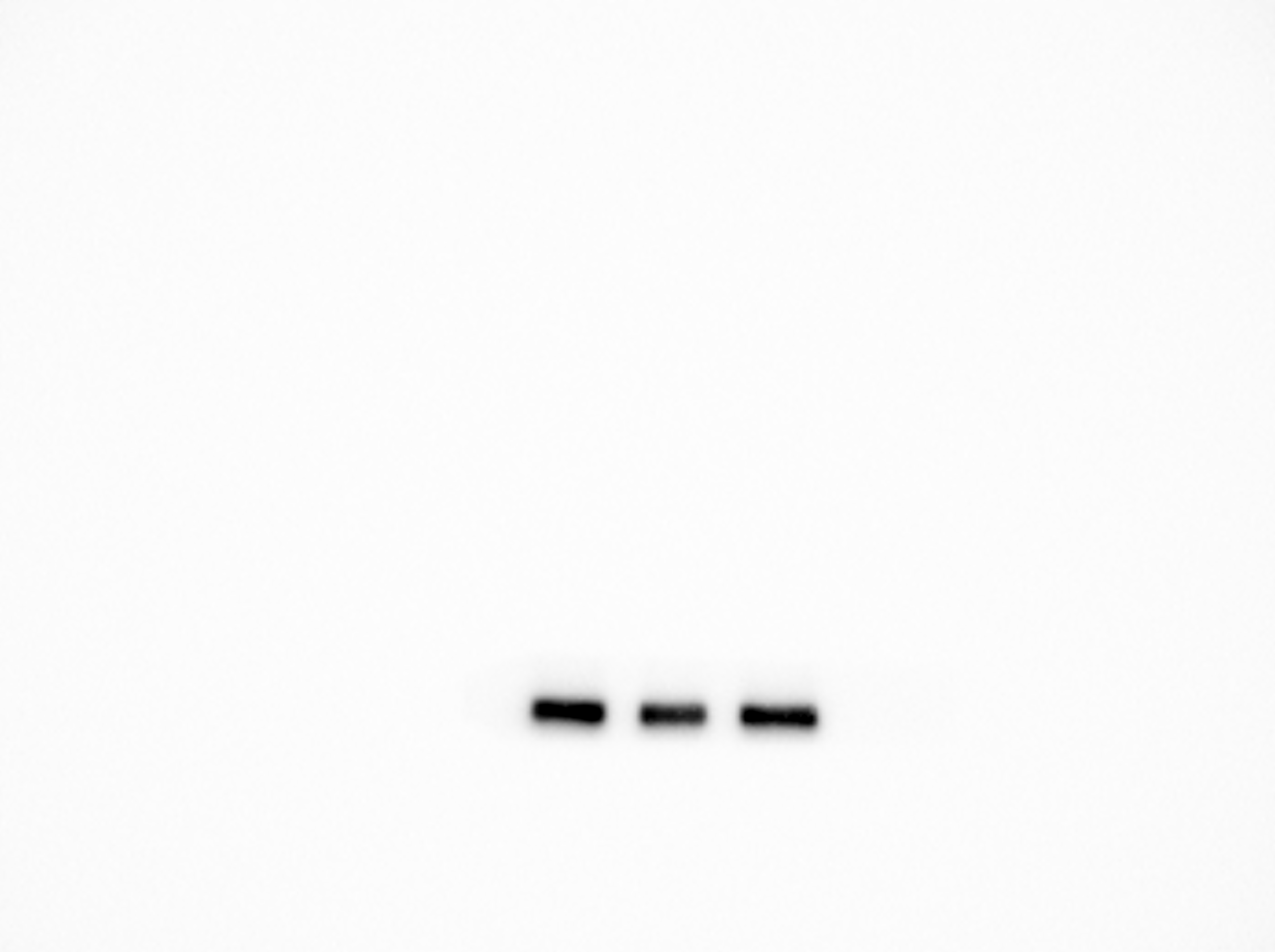

Supplement: Supplementary file 1 [file Data_Sheet_1.ZIP › Fig6A-MEK.tif]

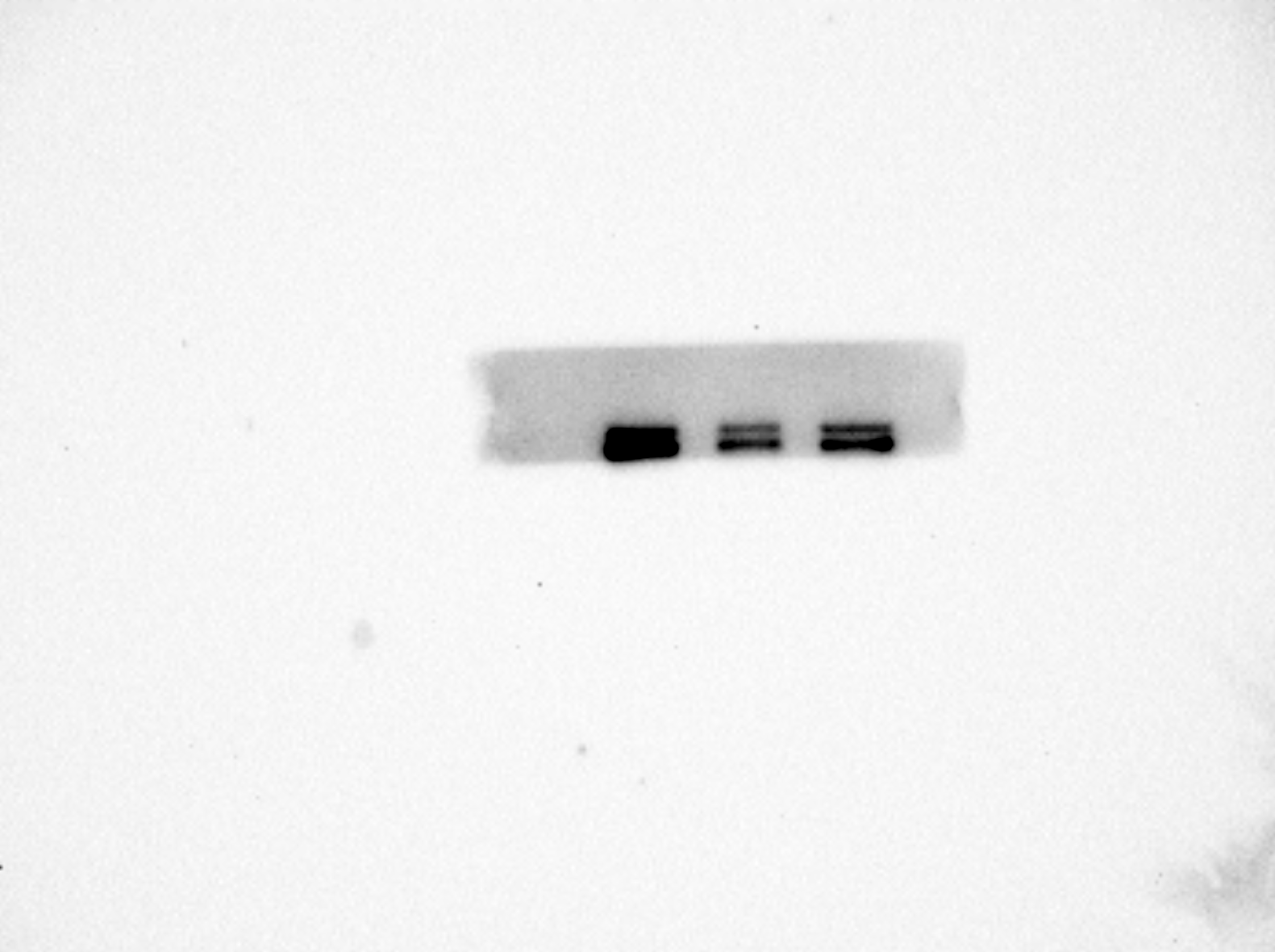

Supplement: Supplementary file 1 [file Data_Sheet_1.ZIP › Fig6A-P-ERK.tif]

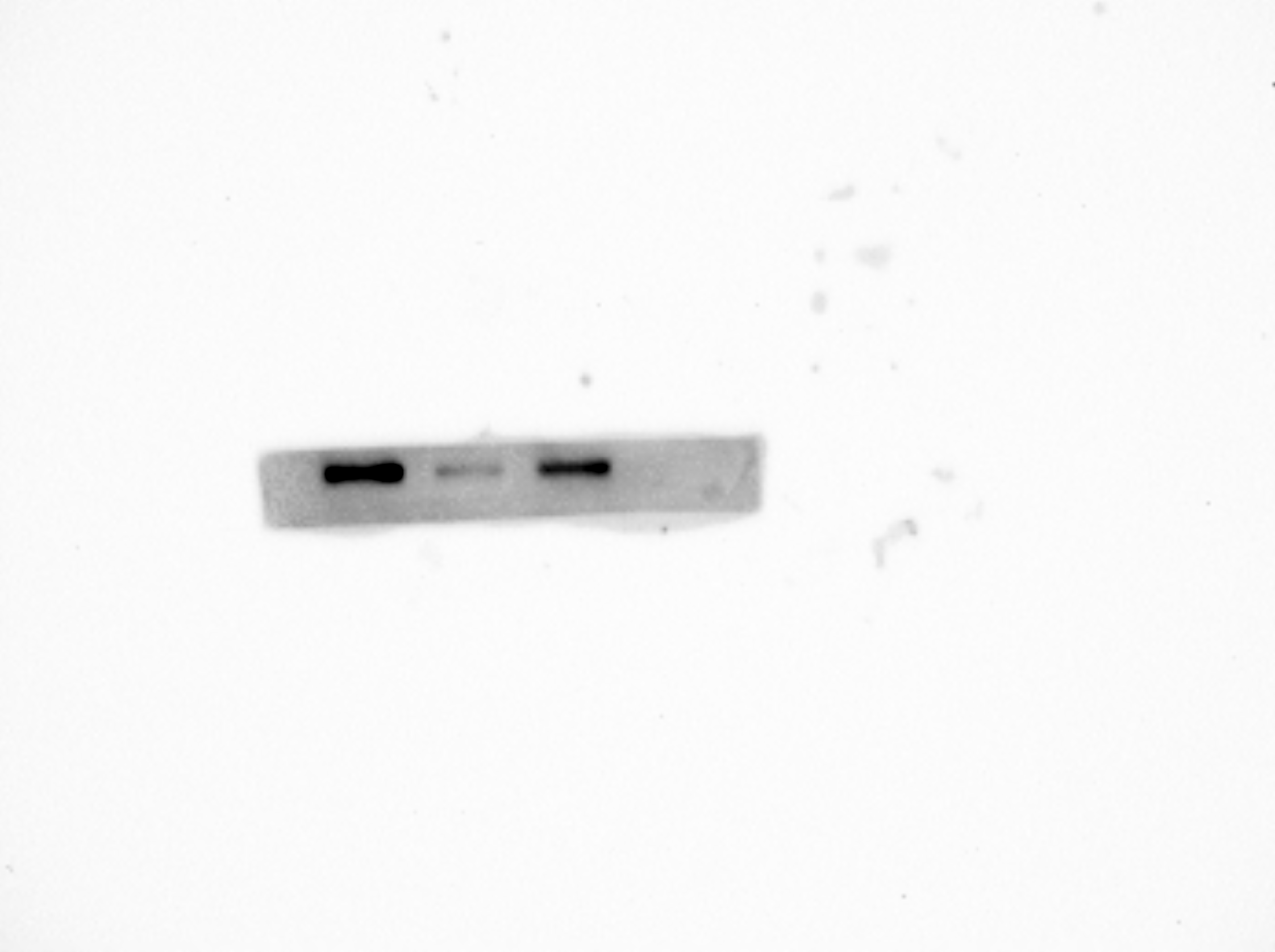

Supplement: Supplementary file 1 [file Data_Sheet_1.ZIP › Fig6A-P-MEK.tif]

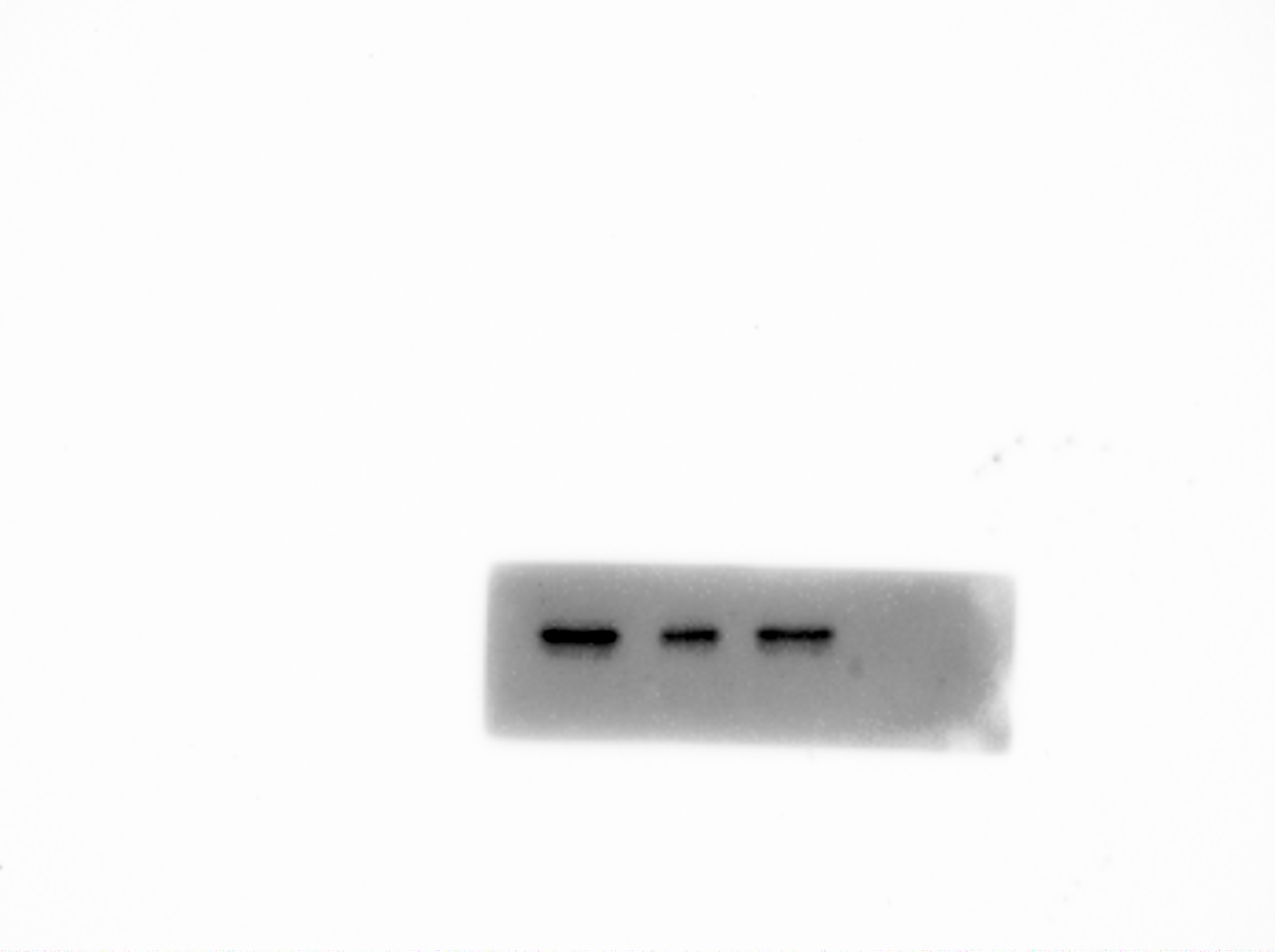

Supplement: Supplementary file 1 [file Data_Sheet_1.ZIP › Fig6A-RAS.tif]

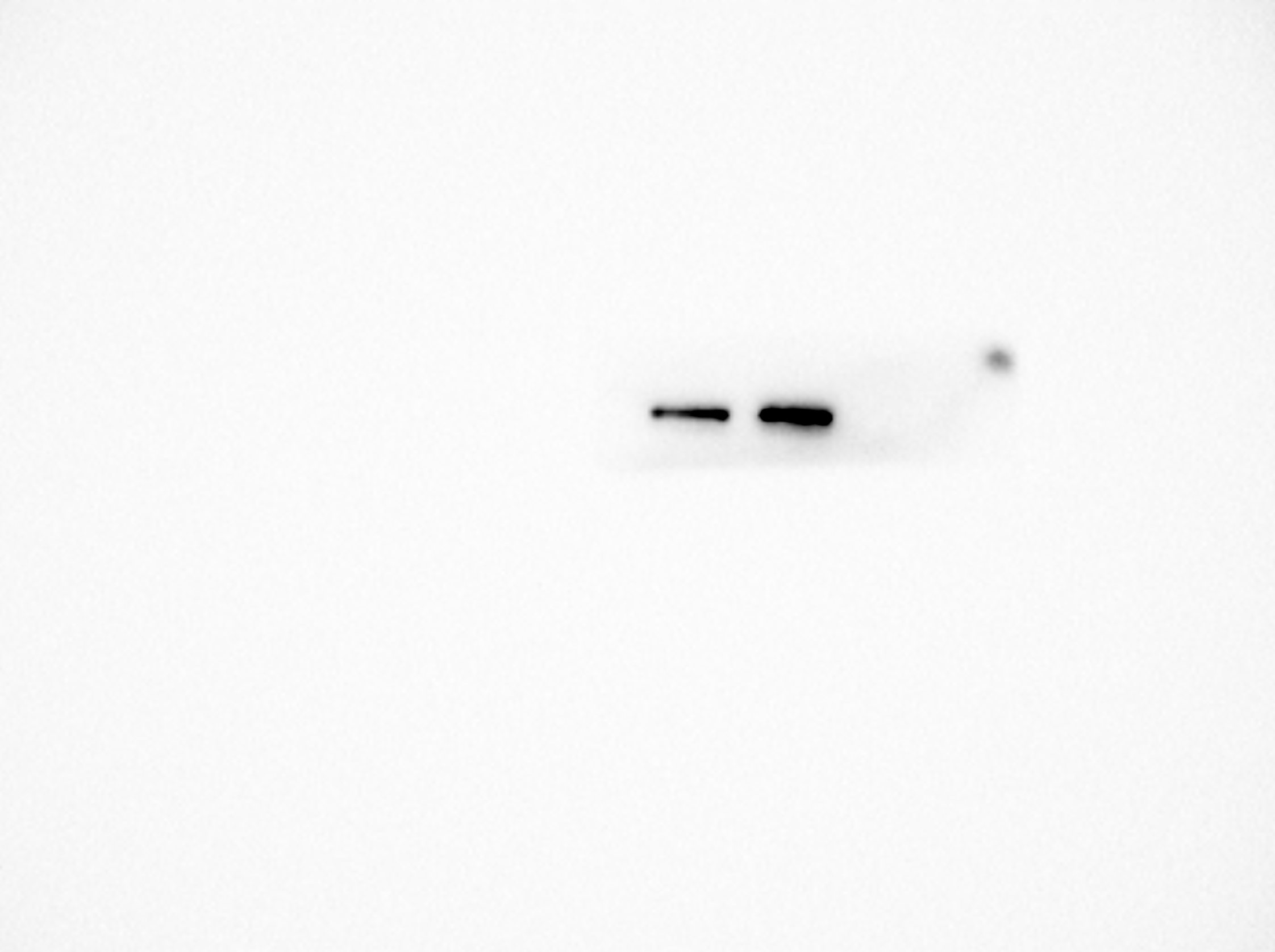

Supplement: Supplementary file 1 [file Data_Sheet_1.ZIP › Fig6B-ANGPT1.tif]

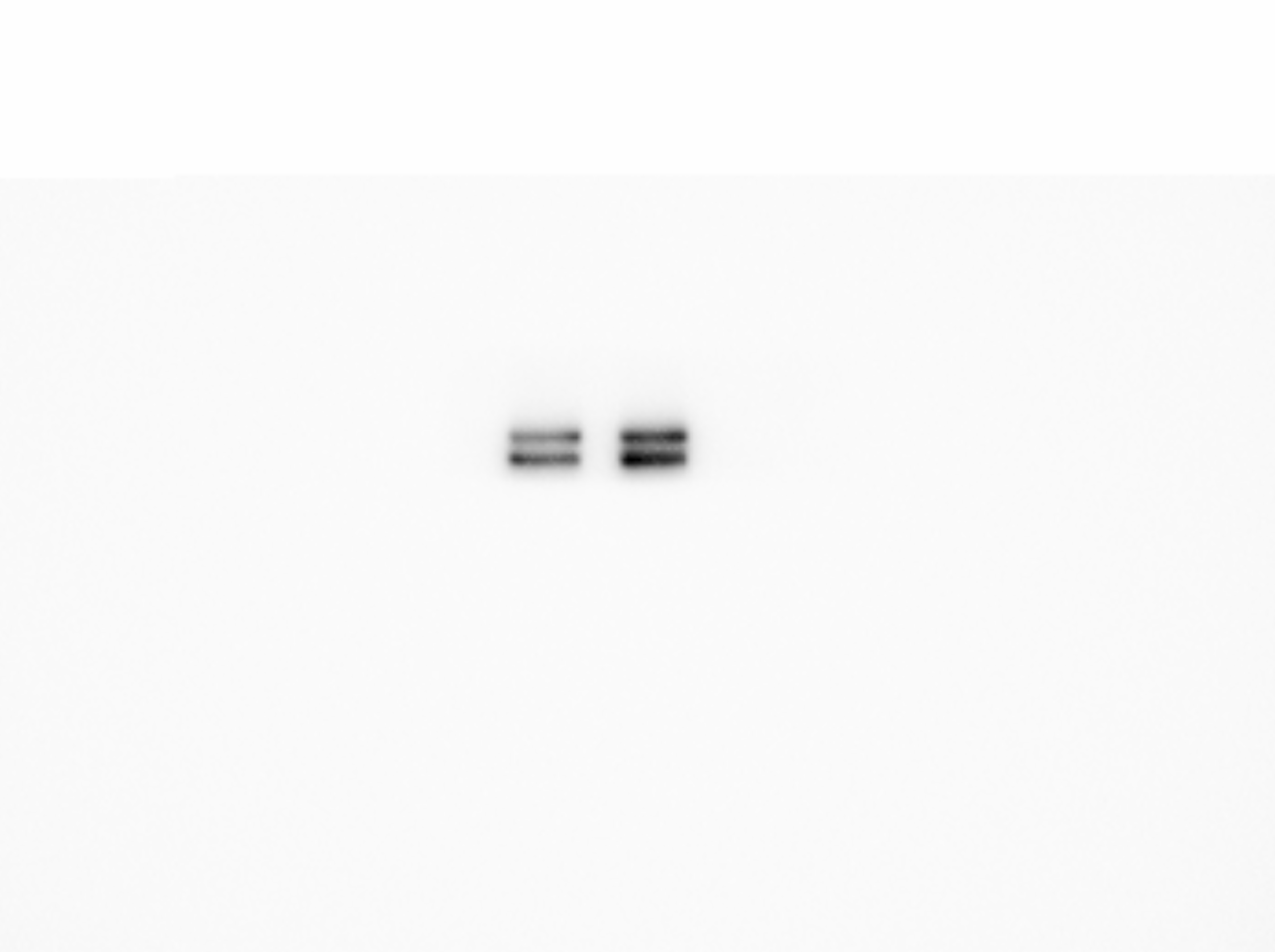

Supplement: Supplementary file 1 [file Data_Sheet_1.ZIP › Fig6B-ERK.tif]

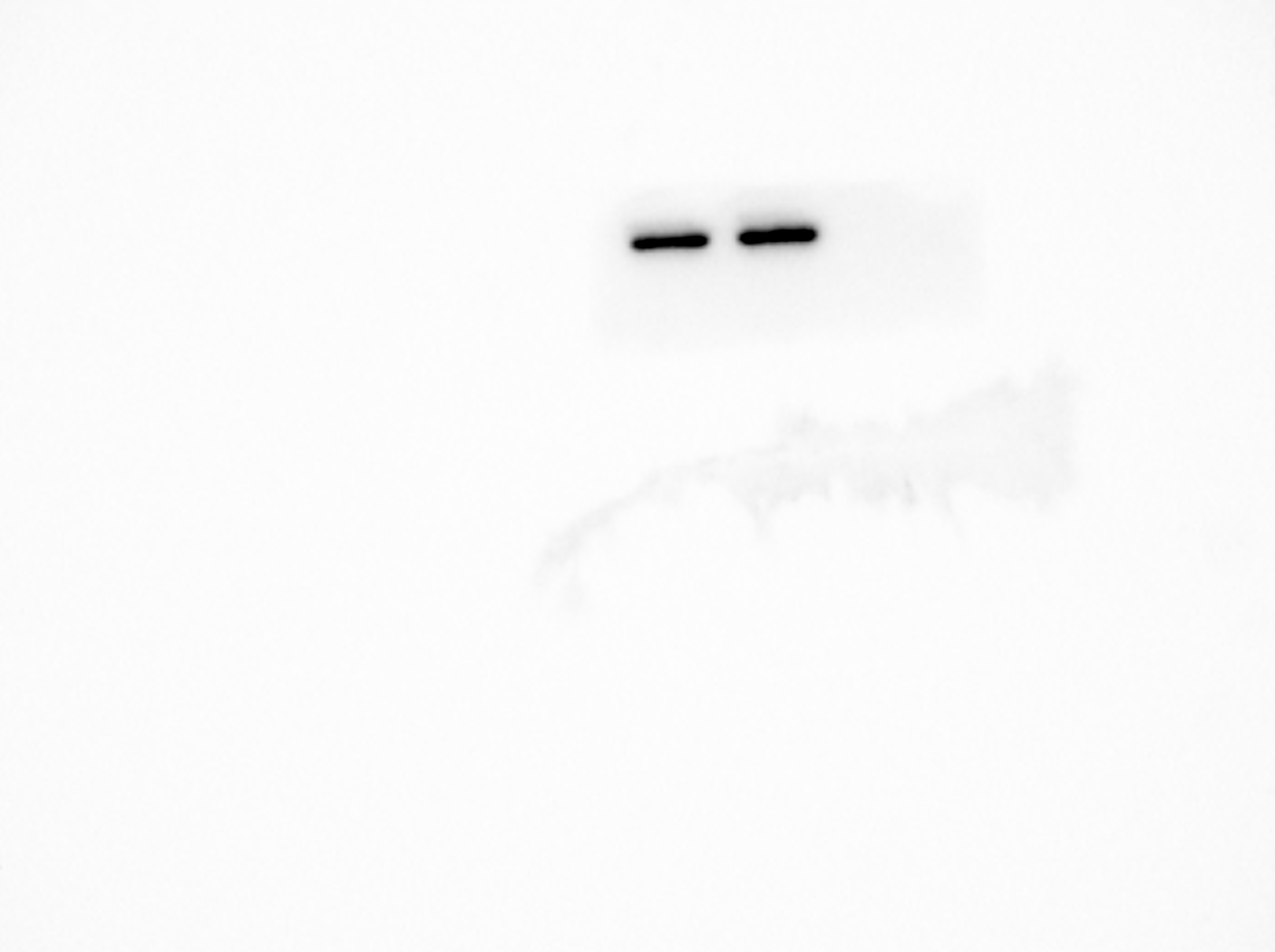

Supplement: Supplementary file 1 [file Data_Sheet_1.ZIP › Fig6B-GAPDH.tif]

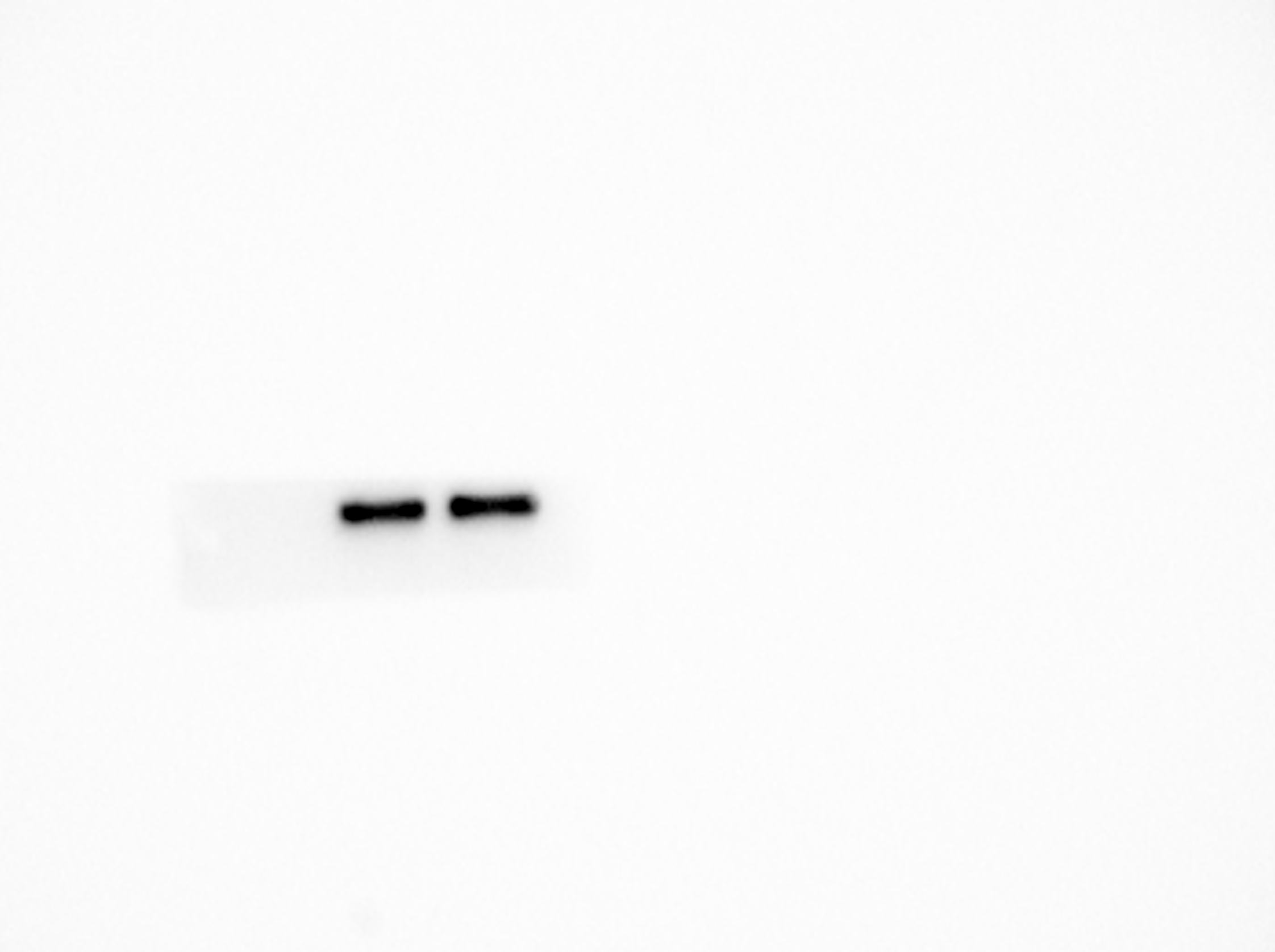

Supplement: Supplementary file 1 [file Data_Sheet_1.ZIP › Fig6B-MEK.tif]

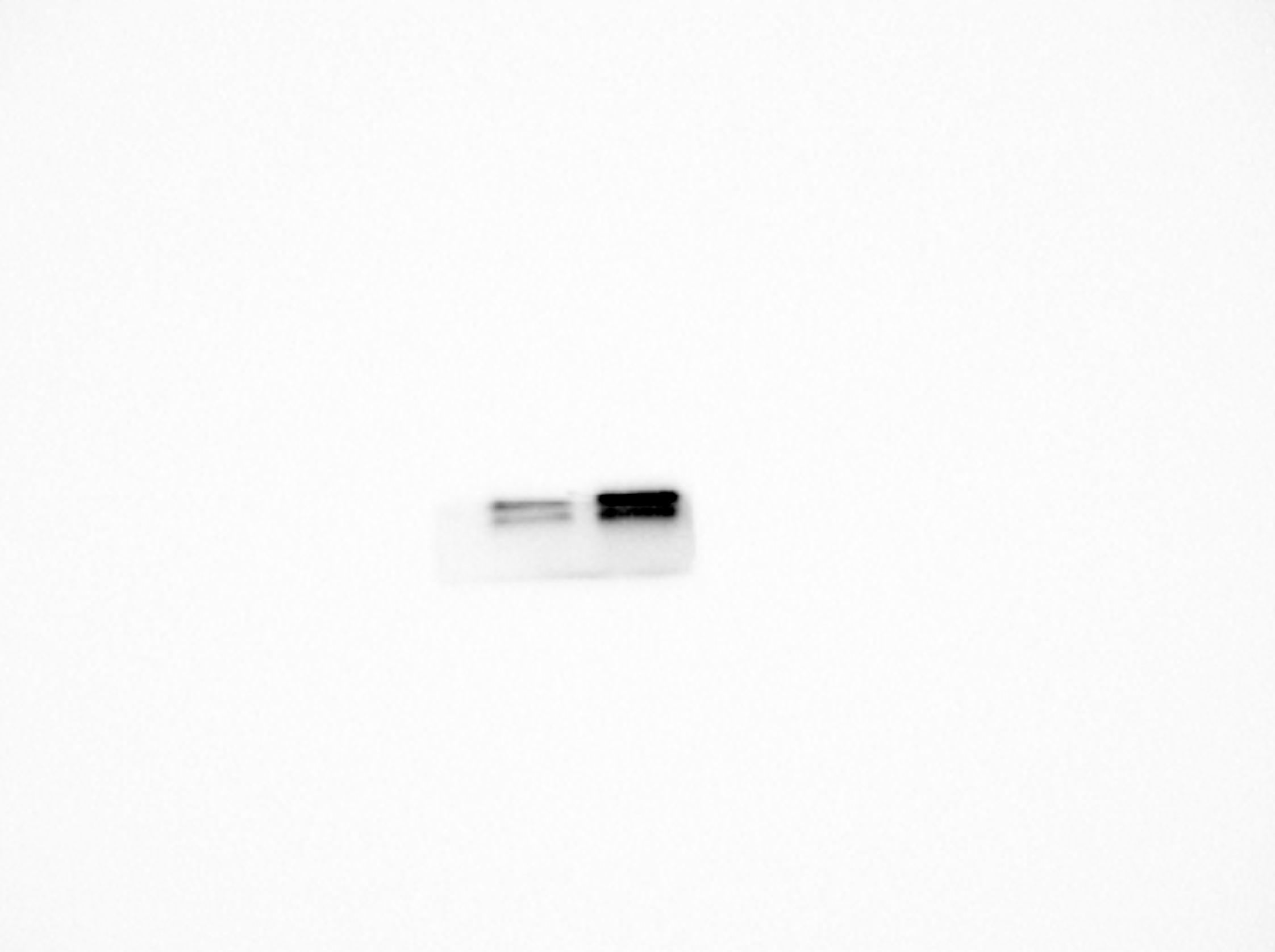

Supplement: Supplementary file 1 [file Data_Sheet_1.ZIP › Fig6B-P-ERK.tif]

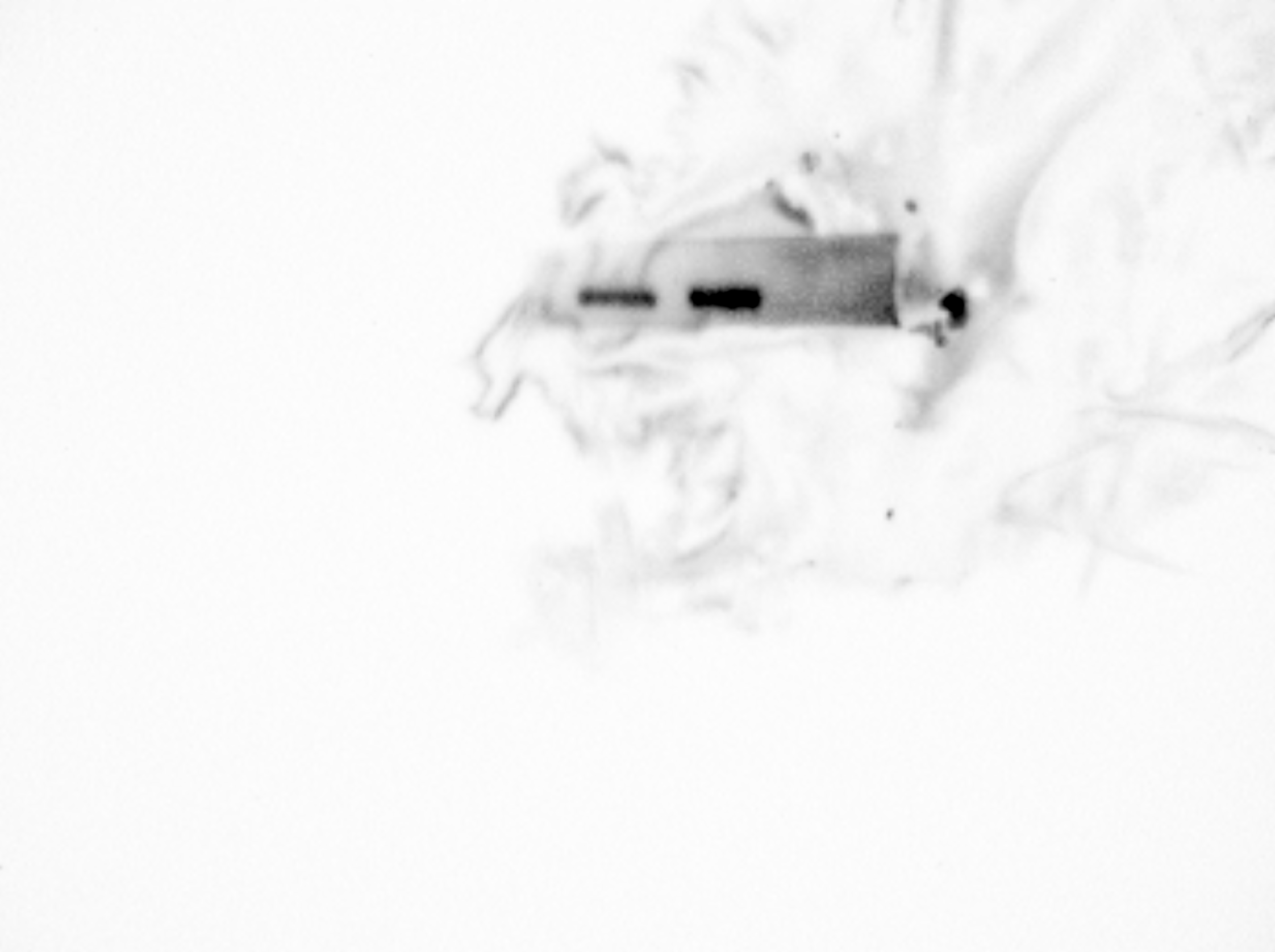

Supplement: Supplementary file 1 [file Data_Sheet_1.ZIP › Fig6B-P-MEK.tif]

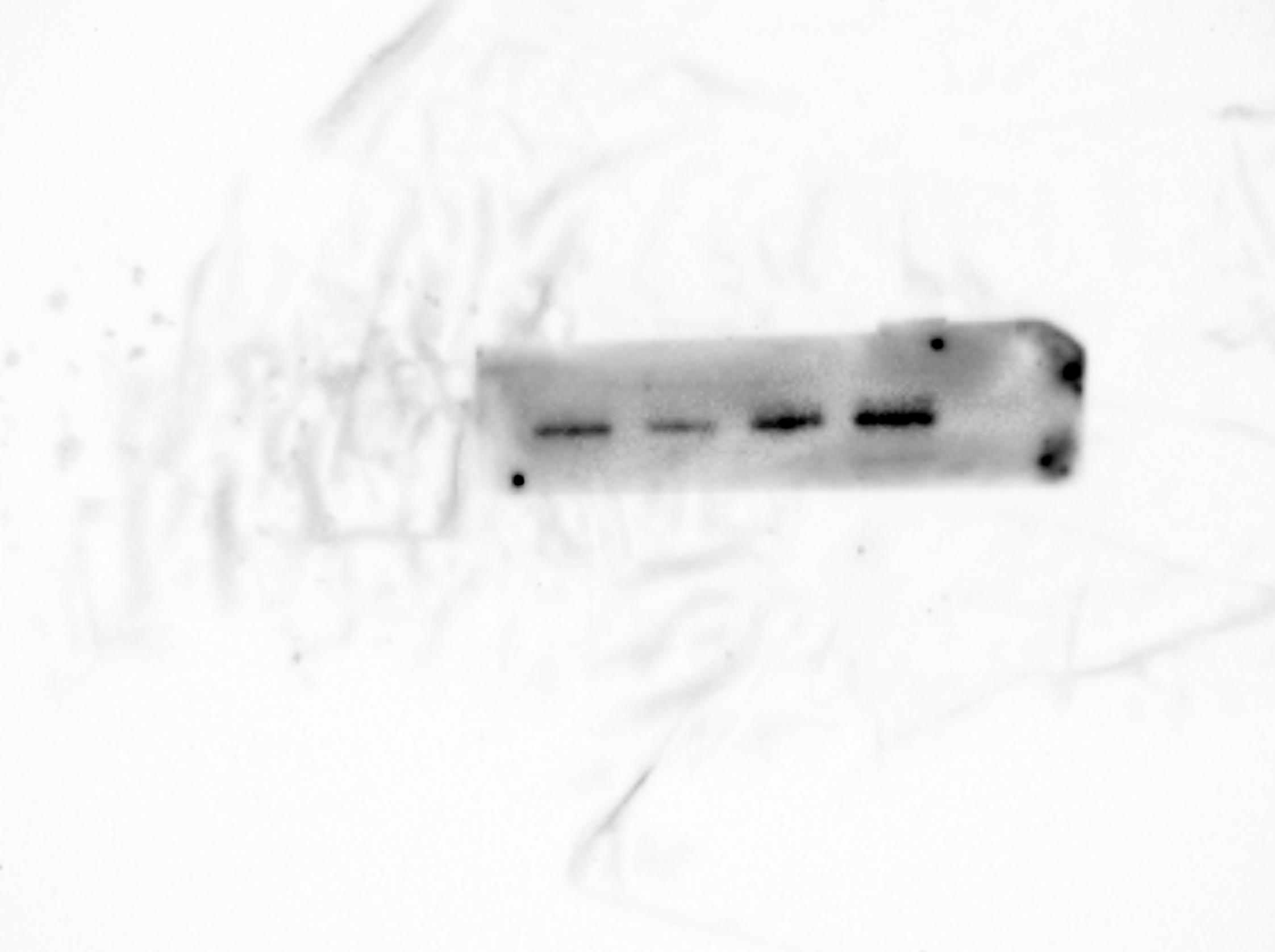

Supplement: Supplementary file 1 [file Data_Sheet_1.ZIP › Fig6B-RAS.tif]

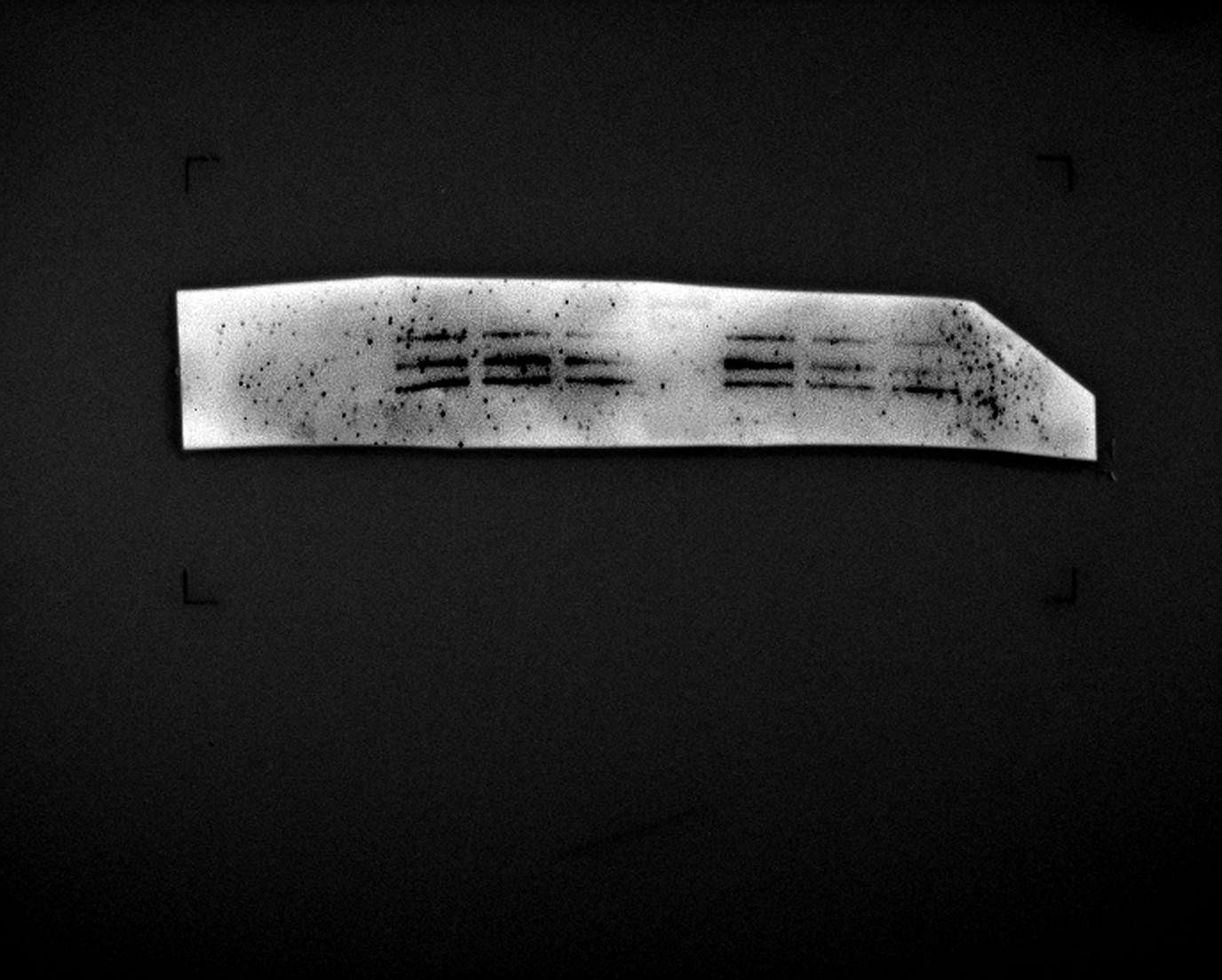

Supplement: Supplementary file 1 [file Data_Sheet_1.ZIP › Fig1-Alix.tif]
